# The one-month impact of influenza infection on patient-reported symptom burden among symptomatic U.S. adults with a test-confirmed illness: a longitudinal study

**DOI:** 10.1186/s12879-026-13950-y

**Published:** 2026-07-09

**Authors:** Tianyan Hu, Laura L. Lupton, Alon Yehoshua, Joseph C. Cappelleri, Meghan B. Gavaghan, Verna L. Welch, Santiago M. C. Lopez, Rachel Brathwaite, Manuela Di Fusco, Xiaowu Sun

**Affiliations:** 1 Pfizer Inc., New York, NY USA; 2 Life Sciences Solutions, CVS Healthspire™, CVS Health® Corporation, Wellesley, MA USA

**Keywords:** Influenza, Symptom burden, Patient-reported data, Outpatient, Prospective, Longitudinal, Repeated survey

## Abstract

**Background:**

Evidence describing the patient-reported symptom burden of influenza infection remains limited. This study assessed prevalence and symptom severity over one-month post-infection during the high-severity 2024–2025 United States (U.S.) season.

**Methods:**

Symptomatic adults ≥ 18 years with positive influenza tests were recruited nationwide from ambulatory care clinic sites in a U.S. national clinic network from 10/24/2024 to 4/24/2025. Participants prospectively completed a symptom survey daily from Days 1 through 7, Day 10, Day 14, and Week 4 after enrollment. Day 1 included retrospective reporting of the worst moment since infection and pre-influenza symptoms (baseline). Prevalence and severity of 12 acute influenza-related symptoms were assessed using a 4-point scale (0 = none, 1 = mild, 2 = moderate, 3 = severe). A composite score was derived by summing ratings across 12 symptoms. Adjusted mixed models for repeated measures estimated the longitudinal impact of influenza infection on symptom burden. Least squares estimates (LSEs) were used to derive mean change and standard error (SE) at each timepoint relative to baseline. Effect size (ES) was calculated using Cohen’s d.

**Results:**

The analysis included 724 adults (mean (standard deviation [SD]) age: 42.0 [13.0] years), 74.2% female, 49.3% vaccinated against influenza, and 27.6% had ≥ 1 comorbidity. On Day 1, the mean composite symptom score was 17.1 (SE: 0.3), with an average of 8.7 total symptoms (SE: 0.1), 5.9 moderate-to-severe (SE: 0.1), and 2.5 severe symptoms (SE: 0.1). At Week 4, there were mean increases of 0.5 points in composite symptom score (SE: 0.2), 0.3 in any symptoms (SE: 0.1), and 0.2 in moderate-to-severe symptoms (SE: 0.1) compared to pre-infection. Severe symptoms persisted through Day 5, with a mean increase of 0.2 (SE: 0.1) on Day 5.

**Conclusion:**

Influenza infection was associated with a high symptom burden that peaked in the first days of illness but extended over four weeks. Severe symptoms decreased over time, becoming moderate or mild, although milder symptoms were still reported at Week 4. These findings highlight the burden of influenza and reinforce the importance of strategies to mitigate this burden including vaccination and other preventive interventions.

**Trial registration:**

The study was first registered on ClinicalTrials.gov (NCT05160636) on 12/15/2021.

**Supplementary Information:**

The online version contains supplementary material available at https://doi.org/10.1186/s12879-026-13950-y.

## Introduction

Seasonal influenza causes substantial morbidity, hospitalization and death among United States (U.S.) adults each year [1]. According to annual estimates from the Centers for Disease Control and Prevention (CDC), influenza has resulted in approximately 9.4–51 million illnesses, 120,000-170,000 hospitalizations, and 6,300 − 52,000 deaths per year over a 15-year period from 2010 to 2025 [1]. Recent post-pandemic seasons (from 2022 to 2023) have shown increasing numbers of influenza illnesses, hospitalizations, and deaths, with the most recent season (2024–2025) recording 51 million illnesses, 710,000 hospitalizations, and 45,000 deaths. Notably, the number of illnesses in 2024–2025 exceeded those observed during the last severe season in 2017–2018, underscoring the substantial ongoing burden.

The infection is commonly associated with respiratory symptoms including cough, sore throat, stuffy or runny nose, and shortness of breath [2]. These are often accompanied by systemic symptoms such as fever, headache, and muscle aches. In some cases, gastrointestinal symptoms including nausea, vomiting, and diarrhea may also occur [2, 3]. Depending on the severity and duration, these symptoms can lead to prolonged discomfort and functional impairment, negatively affecting health-related quality of life, work productivity, and daily activities [4–6]. Collectively, influenza symptoms place a substantial burden on patients and on the broader healthcare system, economy, and society [7].

While influenza infection affects individuals across all age groups, those aged 65 years and older and individuals with underlying conditions experience higher rates of influenza-related hospitalization and death [8–12]. These patterns reflect a disproportionately higher burden of severe outcomes relative to younger, healthier adults [13, 14]. Although disparities in complications are well-documented [8–12], hospitalization rates alone do not capture the full burden of influenza. Because most cases are managed in outpatient settings or at home, repeated assessment of symptoms over time is essential to characterize the course and persistence of illness among non-hospitalized patients. Despite this, few studies have collected longitudinal patient-reported symptom data especially in the outpatient setting, and existing evidence on symptom burden, overall and by age or risk group, is limited [5, 15, 16].

Prior research on influenza symptom burden has key limitations. Despite higher recent rates of influenza [17], few studies have examined longitudinal symptom burden and severity [15, 18, 19], all of which were generated in the pre-pandemic phase [15]. These studies are limited by short follow-up periods (14 days), a focus on feasibility of data collection rather than detailed symptom trajectories [19], and designs with limited generalizability to real-world outpatient settings [15].

For example, a previous cross-sectional study (*n* = 2,374) in acute respiratory illness, across multiple influenza seasons, found that older adults reported lower symptom severity compared with younger adults [20], but it did not capture how symptoms evolved throughout the course of an episode of illness. A human challenge model study, while not generalizable to real-world settings showed symptoms peaked early before gradually declining, with patterns varying by body system. This highlighted the dynamic nature of symptom progression, and the importance of repeated assessments with broad symptom coverage in order to capture the full trajectory of illness [15]. Overall, these limitations highlight the need for real-world, longitudinal data to evaluate longer-term outcomes, such as persistent symptoms including among older adults or those with chronic conditions.

Gathering longitudinal data is especially critical given the CDC’s classification of the 2024–2025 respiratory season as a high severity season, the first high severity season since 2017–2018 [21], based on elevated rates of influenza-like illnesses, and influenza-related hospitalizations and deaths [22]. High severity seasons magnify the clinical burden of influenza, highlighting the urgent need for comprehensive research on symptom trajectories in real-world settings. This includes among older adults and individuals with chronic conditions, whose burden, symptom severity and recovery timelines are not well characterized in high severity seasons.

To address this gap, this study used longitudinal patient-reported data collected during the 2024–2025 high-severity U.S. influenza season. The study objective was to examine prevalence, severity, duration, and trajectory of influenza symptoms over 4 weeks following test-confirmed infection in symptomatic U.S. adults overall, and specifically in clinically relevant high-risk groups.

## Methods

### Study design and participants

This was a prospective, noninterventional, repeated-measures study conducted during the Northern Hemisphere 2024–2025 U.S. influenza season (ClinicalTrials.gov identifier: NCT05160636). Adults with a positive influenza test at national ambulatory care clinic network sites in the U.S., were identified from 10/24/2024 to 4/24/2025. The national clinic network included 823 ambulatory care clinics across 31 U.S. states providing broad geographic coverage. Within 48 h of the study team receiving the results, individuals were emailed an invitation to participate. Eligible participants provided electronic informed consent and completed the first survey on Day 1.

Participants were required to meet all of the following criteria: 1) age ≥ 18 years; 2) ≥ 1 self-reported symptom/s on the screening questionnaire; 3) symptom onset 0–4 days before the positive test; 4) a positive rapid influenza diagnostic tests or rapid molecular assays for influenza infection for influenza at ambulatory care clinic sites in a U.S. national clinic network; 5) provided electronic informed consent; 6) able to complete questionnaires in English; and 7) access to internet and a device. Exclusion criteria included receipt of influenza vaccination within 14 days of symptom onset or prior test-confirmed influenza within 30 days of completing screening.

Participants who provided informed consent were sent follow-up surveys to gather information on socio-demographic and clinical characteristics, as well as prevalence and severity of symptoms. Participants received up to $160 in compensation for completing all study surveys during the 1-month follow-up period; this was reviewed and approved by the Institutional Review Board (IRB).

### Socio-demographic and clinical characteristics

Demographic characteristics collected included age, self-reported sex and race/ethnicity, and U.S. geographic region. The Social Vulnerability Index (SVI) was assigned as an indicator of communities’ vulnerability [23]. The SVI ranges from 0 (least vulnerable) to 1 (most vulnerable) [23]. Participants self-reported comorbid conditions that aligned with the CDC definition of being at increased risk for influenza complications (Table S1, Additional File 1) [13]. Participants also self-reported 2024–2025 influenza vaccine status, time since vaccination, use of antiviral treatment (oseltamivir phosphate (Tamiflu^®^), baloxavir marboxil (Xofluza^®^), zanamivir (Relenza^®^), or other antiviral medications) for the current influenza illness, as well as pregnancy and smoking status.

### Prevalence and severity of acute symptoms

**Fig. 1:**
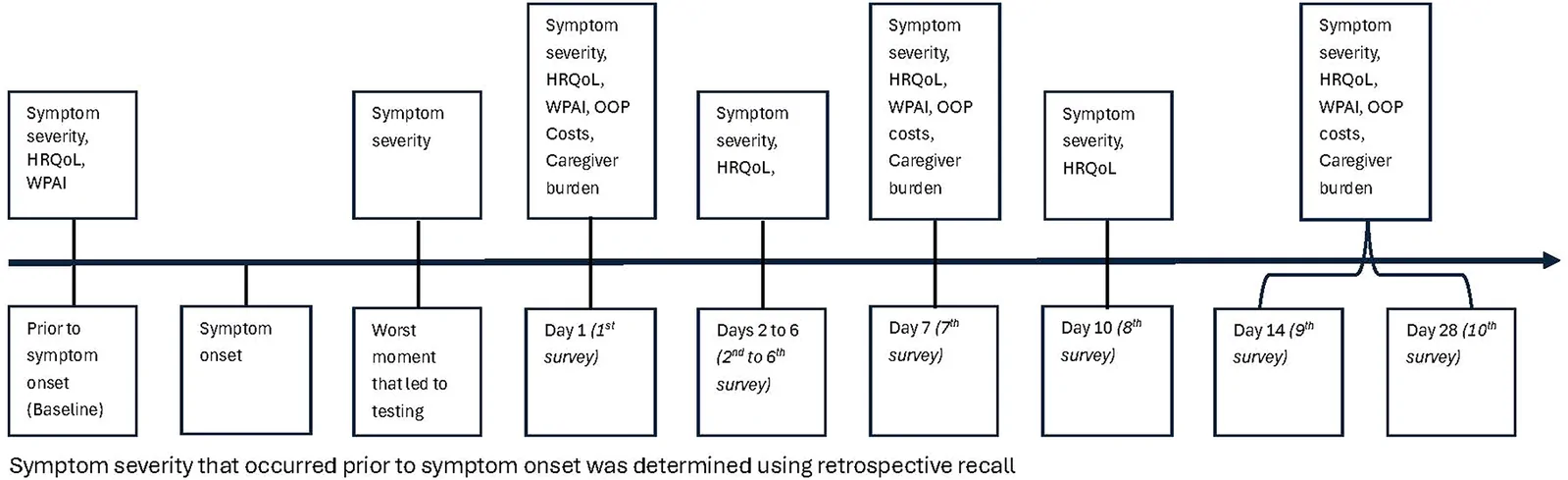
Study design and process flow. HRQoL: Health-related quality of life measured using EQ-5D-5L utility index (UI) and visual analogue scale (VAS); WPAI: Work Productivity and Activity Impairment; OOP: out-of-pocket

The prevalence and severity of 12 acute symptoms associated with influenza infection identified by the CDC [24] and previous literature were assessed [25]. Symptoms were grouped into systemic (e.g., feverish, chills/shivering, muscle/body aches, headache, fatigue), respiratory (e.g., shortness of breath, runny/stuffy nose, cough, sore throat), or gastrointestinal (e.g., nausea, vomiting, diarrhea). Participants rated all systemic and respiratory symptoms as well as nausea using a 4-point ordinal scale (0 = none, 1 = mild, 2 = moderate, or 3 = severe). Participants reported the frequency of vomiting and diarrhea using an ordinal categorical scale with response options of 0 times, 1–2 times, 3–4 times, and 5 or more times. Symptom assessment occurred at multiple time points: at baseline, referring to the pre-infection period and captured through retrospective reports of symptoms and health status before the influenza illness began; at the worst moment since symptom onset; and at the worst moment during the past 24 h at enrollment (Day 1) [20]. All Day 1 measures (baseline and worst moment) were collected retrospectively. The ‘worst moment since symptom onset’ reflects the self-identified peak symptom severity at any point between symptom onset and completion of the Day 1 survey, which occurred after test-confirmation. Follow-up assessments were scheduled on Days 2–7, 10, 14, and Week 4 (Fig. 1). The study evaluated the prevalence of influenza-related symptoms across three nested severity thresholds to capture different levels of symptom intensity. Any symptoms reflect the presence of any reported symptoms regardless of severity, moderate-to-severe reflects more substantial symptoms, and severe isolates only the most intense symptoms. Symptom severity has not been comprehensively assessed in previous literature, making this a unique contribution of this study. A composite score (0–36) was derived by summing ratings across all 12 symptoms, with higher values indicating greater burden [20]. Internal consistency of the score was high (Cronbach’s α = 0.88).

### Statistical analysis

Baseline characteristics and outcomes were summarized using descriptive statistics. Continuous variables were summarized as means and standard deviations (SD), and categorical variables as counts and percentages.

Mixed models for repeated measures (MMRM) were used to estimate longitudinal changes in symptom outcomes [26]. Models included time (categorical), high-risk status, self-reported sex and race/ethnicity, region, SVI, antiviral use, influenza vaccination status, and baseline scores. Interaction terms were specified for time with high-risk status, and time with vaccination status, with an unstructured covariance matrix for repeated measures. Changes in composite symptom scores, and the mean numbers of any symptoms, moderate-to-severe, and severe symptoms (at the worst moment, and Day 1 through Week 4), were estimated relative to baseline using these covariate-adjusted models. For each MMRM, least squares mean (LS mean) and standard error (SE) were derived. Model-based t-tests were used to assess whether mean changes from baseline were statistically different from zero. P-values < 0.05 were considered statistically significant, without adjustment for multiplicity. The within-cohort effect size (ES) (Cohen’s d) [27, 28] was determined by estimating the mean change from prior to infection to follow-up, divided by the SD of change scores [29]. These standardized effect sizes were interpreted using Cohen’s conventions, with threshold values of 0.2 to < 0.5, 0.5 to < 0.8, and ≥ 0.8 indicating “small,” “medium,” and “large” effects, respectively [27, 29]. MMRM analyses assumed data were missing at random (MAR); missing data were not imputed.

Additionally, to aid interpretation, visual summaries of longitudinal trends were plotted for: (1) prevalence of any symptoms; (2) prevalence of moderate-to-severe symptoms; (3) prevalence of severe symptoms; (4) mean composite symptom score; (5) mean number of any symptoms (mild-to-severe); (6) mean number of moderate-to-severe symptoms; and (7) mean number of severe symptoms.

All analyses were conducted in SAS Version 9.4 (SAS Institute, Cary, NC). Reporting followed the Strengthening the Reporting of Observational Studies in Epidemiology (STROBE) guideline for cohort studies [30].

### Sensitivity analysis: High-risk group

As a way to assess symptom burden among high-risk population, the following analyses were conducted. First, adults ≥ 65 years, adults < 65 years with ≥ 1 CDC-recognized comorbidity, and pregnant people were included, consistent with CDC definitions for increased influenza-related risk [13]. Secondly, the analysis was restricted to adults < 65 years with comorbidity or pregnancy isolating the effect of comorbidity independent of age. Finally, given overlapping respiratory risk profiles and symptoms, an exploratory analysis evaluated adults at high-risk for coronavirus disease 2019 (COVID-19) using CDC criteria [31], which differ from the influenza high-risk definition [13] by including mental health conditions and smoking and excluding blood disorders [32].

## Results

### Baseline socio-demographic and clinical characteristics in the total population

**Fig. 2:**
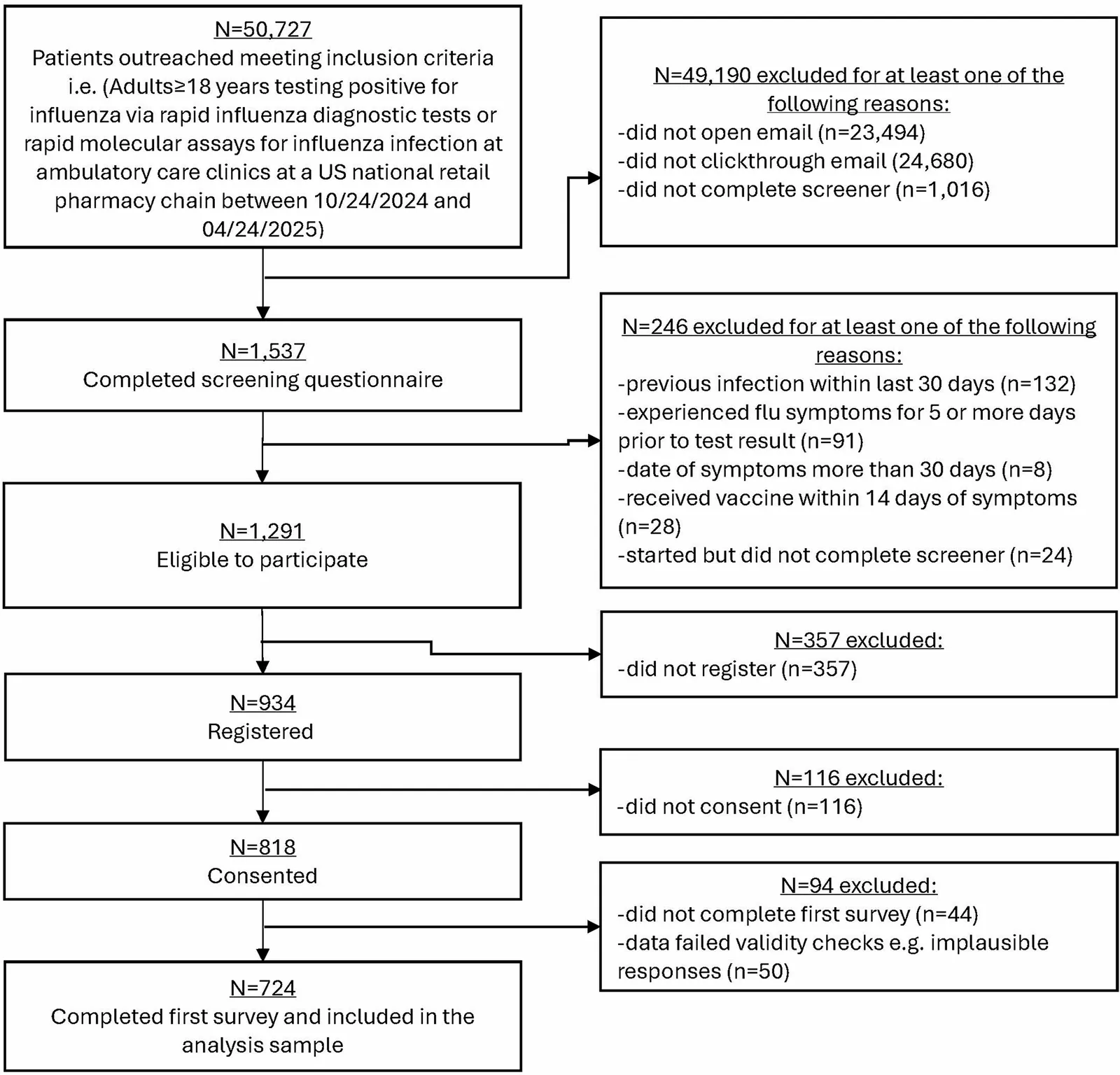
Study participant flow chart

Figure 2 provides details on the study participant flow. A total of 724 eligible adults were included in the analysis sample. The mean (standard deviation (SD) age was 42.0 (13.0) years, and 74.2% were female (Table 1). The majority (80.9%) self-identified as White, followed by Hispanic/Latino (7.2%) and Black/African American (4.8%). Most resided in the South (46.7%) and Midwest (30.5%) regions and lived in less vulnerable communities (mean SVI = 0.3). 49% received the 2024–2025 influenza vaccine and 85.1% reported receiving antiviral treatment. Approximately 27.6% of individuals had at least one comorbidity, most commonly, obesity (14.4%), chronic lung conditions (7.0%), and endocrine disorders (4.6%). Nine people (1.2%) were pregnant. The median time from symptom onset to Day 1 was three days (interquartile range (IQR): 2–4). The median time from vaccination to enrollment was 106 days (IQR: 82–130). Participants completed a median of 10 surveys (IQR: 7–10). Retention rates are presented in Table 2. By Week 4, 224 participants dropped out, yielding a 69.1% retention rate (Table 2).

**Table 1:** Baseline demographic and clinical characteristics of study participants in total population and high-risk subgroup

	Total population (*N* = 724)	Influenza high-risk group (all ages) (*N* = 225)
Age, years, mean (SD)	42.0 (13.0)	47.2 (14.6)
Self-reported sex, n (%)		
Female	537 (74.2)	168 (74.7)
Male	187 (25.8)	57 (25.3)
Race/Ethnicity, n (%)		
White	586 (80.9)	191 (84.9)
Black or African American	35 (4.8)	9 (4.0)
Hispanic or Latino	52 (7.2)	15 (6.7)
Asian	24 (3.3)	3 (1.3)
Other	27 (3.7)	7 (3.1)
US Region of residence, n (%)		
Northeast	128 (17.7)	43 (19.1)
South	338 (46.7)	101 (44.9)
Midwest	221 (30.5)	70 (31.1)
West	33 (4.6)	10 (4.4)
Other\Unknown	4 (0.6)	1 (0.4)
Employment status, n (%)		
Yes	593 (81.9)	168 (74.7)
Social Vulnerability Index ^a^, mean (SD)	0.32 (0.19)	0.33 (0.19)
Social Vulnerability Index category, n (%)		
< 0.25	315 (43.5)	91 (40.4)
≥ 0.25 and < 0.5	293 (40.5)	96 (42.7)
≥ 0.5 and < 0.75	90 (12.4)	29 (12.9)
≥ 0.75	25 (3.5)	8 (3.6)
Anti-viral use, n (%)	616 (85.1)	193 (85.8)
Received the 2024–2025 influenza vaccine, n (%)	357 (49.3)	122 (54.2)
Pregnant, n (%)		
No	520 (71.8)	145 (64.4)
Yes	9 (1.2)	9 (4.0)
Not applicable	195 (26.9)	71 (31.6)
Underlying health conditions, n (%)		
Any current/active cancers or malignancies (other than skin cancer)	5 (0.7)	5 (2.2)
Cerebrovascular disease	8 (1.1)	8 (3.5)
Chronic kidney disease	3 (0.4)	3 (1.3)
Chronic lung conditions	51 (7.0)	51 (22.7)
Chronic liver disease	11 (1.5)	11 (4.9)
Endocrine disorders	33 (4.6)	33 (14.7)
Heart conditions	21 (2.9)	21 (9.3)
Obesity: BMI > 40	104 (14.4)	104 (46.2)
Weakened immune system/ immunocompromised	17 (2.3)	17 (7.6)
Blood disorders	5 (0.7)	5 (2.2)
Active Tuberculosis	0 (0.0)	0 (0.0)
Number of comorbidities ^b^, mean (SD)	0.36 (0.66)	1.15 (0.69)
≥ 1 comorbidity ^b^, n (%)	200 (27.6)	200 (88.9)
≥ 1 comorbidity or pregnant, n (%)	208 (28.7)	208 (92.4)
Time from symptom onset to Day 1, median (IQR)	3 (2–4)	3 (3–4)
Number of surveys completed, median (IQR)	10 (7–10)	10 (7–10)
Time from vaccination to enrollment, median (IQR)	106 (82–130)	115 (88–142)

SD=standard deviation; BMI=body mass index; IQR=interquartile range

^a^SVI is a score that ranges from 0 to 1. Higher values correspond to higher vulnerability

^b^Derived using high-risk criteria for influenza [13]

High-risk population defined as adults ≥ 65 years or adults < 65 years with ≥ 1 CDC-recognized comorbidity, and pregnant women

**Table 2:** Summary of least-square estimates ^a^ of mean change in symptom measures from prior to infection

Time	Retention rate *n* (%)	Composite acute symptom score		Number of any symptoms (mild to severe)		Number of moderate/severe acute symptoms		Number of severe acute symptoms	
		LSE (SE)	ES ^b^	LSE (SE)	ES ^b^	LSE (SE)	ES ^b^	LSE (SE)	ES ^b^
Total population									
Prior to infection ^c^	724 (100.0)	1.2 (1.6)		1.1 (1.4)		0.1 (0.3)		0.0 (0.1)	
Worst Moment	724 (100.0)	**18.8 (0.3)** ^**d**^	3.24	**8.0 (0.1)** ^**d**^	3.95	**7.0 (0.1)** ^**d**^	2.96	**3.8 (0.1)** ^**d**^	1.45
Day 1	724 (100.0)	**15.9 (0.3)** ^**d**^	2.60	**7.6 (0.1)** ^**d**^	3.32	**5.8 (0.1)** ^**d**^	2.16	**2.5 (0.1)** ^**d**^	1.08
Day 2	607 (83.8)	**8.2 (0.3)** ^**d**^	1.58	**5.1 (0.1)** ^**d**^	1.94	**2.5 (0.1)** ^**d**^	1.07	**0.5 (0.1)** ^**d**^	0.47
Day 3	602 (83.1)	**5.6 (0.2)** ^**d**^	1.30	**3.9 (0.1)** ^**d**^	1.53	**1.4 (0.1)** ^**d**^	0.78	**0.3 (0.1)** ^**d**^	0.37
Day 4	586 (80.9)	**4.3 (0.2)** ^**d**^	1.11	**3.1 (0.1)** ^**d**^	1.29	**1.0 (0.1)** ^**d**^	0.67	**0.2 (0.1)** ^**d**^	0.31
Day 5	575 (79.4)	**3.6 (0.2)** ^**d**^	0.92	**2.6 (0.1)** ^**d**^	1.08	**0.8 (0.1)** ^**d**^	0.55	**0.2 (0.1)** ^**d**^	0.24
Day 6	565 (78.0)	**2.9 (0.2)** ^**d**^	0.83	**2.1 (0.1)** ^**d**^	0.92	**0.7 (0.1)** ^**d**^	0.50	0.1 (0.1)	0.29
Day 7	562 (77.6)	**2.6 (0.2)** ^**d**^	0.82	**1.9 (0.1)** ^**d**^	0.84	**0.5 (0.1)** ^**d**^	0.52	0.1 (0.1)	0.25
Day 10	522 (72.1)	**2.1 (0.2)** ^**d**^	0.69	**1.6 (0.1)** ^**d**^	0.71	**0.4 (0.1)** ^**d**^	0.43	0.1 (0.1)	0.26
Day 14	515 (71.1)	**1.3 (0.2)** ^**d**^	0.51	**1.0 (0.1)** ^**d**^	0.49	**0.2 (0.1)** ^**d**^	0.29	0.1 (0.1)	0.28
Week 4	500 (69.1)	**0.5 (0.2)** ^**d**^	0.24	**0.3 (0.1)** ^**d**^	0.18	**0.2 (0.1)** ^**d**^	0.25	0.0 (0.1)	0.30
Influenza high-risk group (all ages)									
Prior to infection ^c^	225 (100.0)	1.5 (1.8)		1.4 (1.6)		0.1 (0.4)		0.0 (0.1)	
Worst Moment	225 (100.0)	**18.7 (0.4)** ^**d**^	3.09	**8.0 (0.2)** ^**d**^	3.66	**7.0 (0.2)** ^**d**^	2.79	**3.8 (0.1)** ^**d**^	1.41
Day 1	225 (100.0)	**15.8 (0.4)** ^**d**^	2.48	**7.5 (0.2)** ^**d**^	3.12	**5.8 (0.2)** ^**d**^	2.04	**2.5 (0.1)** ^**d**^	1.08
Day 2	192 (85.3)	**8.5 (0.4)** ^**d**^	1.54	**5.3 (0.2)** ^**d**^	1.87	**2.7 (0.2)** ^**d**^	1.11	**0.6 (0.1)** ^**d**^	0.44
Day 3	191 (84.9)	**6.0 (0.3)** ^**d**^	1.30	**4.1 (0.2)** ^**d**^	1.50	**1.6 (0.1)** ^**d**^	0.85	**0.3 (0.1)** ^**d**^	0.42
Day 4	186 (82.7)	**4.8 (0.3)** ^**d**^	1.13	**3.4 (0.2)** ^**d**^	1.30	**1.3 (0.1)** ^**d**^	0.71	**0.2 (0.1)** ^**d**^	0.40
Day 5	184 (81.8)	**4.1 (0.3)** ^**d**^	0.90	**2.9 (0.2)** ^**d**^	1.07	**1.0 (0.1)** ^**d**^	0.59	**0.3 (0.1)** ^**d**^	0.26
Day 6	180 (80.0)	**3.4 (0.3)** ^**d**^	0.83	**2.4 (0.2)** ^**d**^	0.92	**0.8 (0.1)** ^**d**^	0.52	0.2 (0.1)	0.36
Day 7	181 (80.4)	**3.1 (0.3)** ^**d**^	0.88	**2.3 (0.2)** ^**d**^	0.91	**0.7 (0.1)** ^**d**^	0.58	0.1 (0.1)	0.32
Day 10	173 (76.9)	**2.5 (0.3)** ^**d**^	0.82	**1.9 (0.2)** ^**d**^	0.80	**0.5 (0.1)** ^**d**^	0.54	0.1 (0.1)	0.32
Day 14	171 (76.0)	**1.6 (0.2)** ^**d**^	0.60	**1.3 (0.2)** ^**d**^	0.59	**0.3 (0.1)** ^**d**^	0.32	0.1 (0.1)	0.42
Week 4	165 (73.3)	**0.7 (0.2)** ^**d**^	0.29	**0.5 (0.2)** ^**d**^	0.27	**0.2 (0.1)** ^**d**^	0.23	0.1 (0.1)	0.62

LSE=least square estimate; SE=standard error; ES=effect size

^a^ Least square estimates based on mixed models for repeated measures, which include covariates of time, high-risk status, sex, race/ethnicity, region, Social Vulnerability Index, antiviral use, receipt of influenza vaccine, baseline outcome score, and interaction terms between time and high-risk status, as well as time and influenza vaccine receipt, with unstructured correlation matrix for repeated measures

^b^ ES refers to within-cohort effect size, was calculated as the least square estimate of mean change scores divided by the observed standard deviation of change scores from prior to infection to follow-up

^c^ Mean (standard deviation) of scores prior to infection reflect observed values and were not derived using the least squares model estimates

^d^*P* < 0.05 considered statistically significant

### Prevalence of acute symptoms at each timepoint in total population

**Fig. 3:**
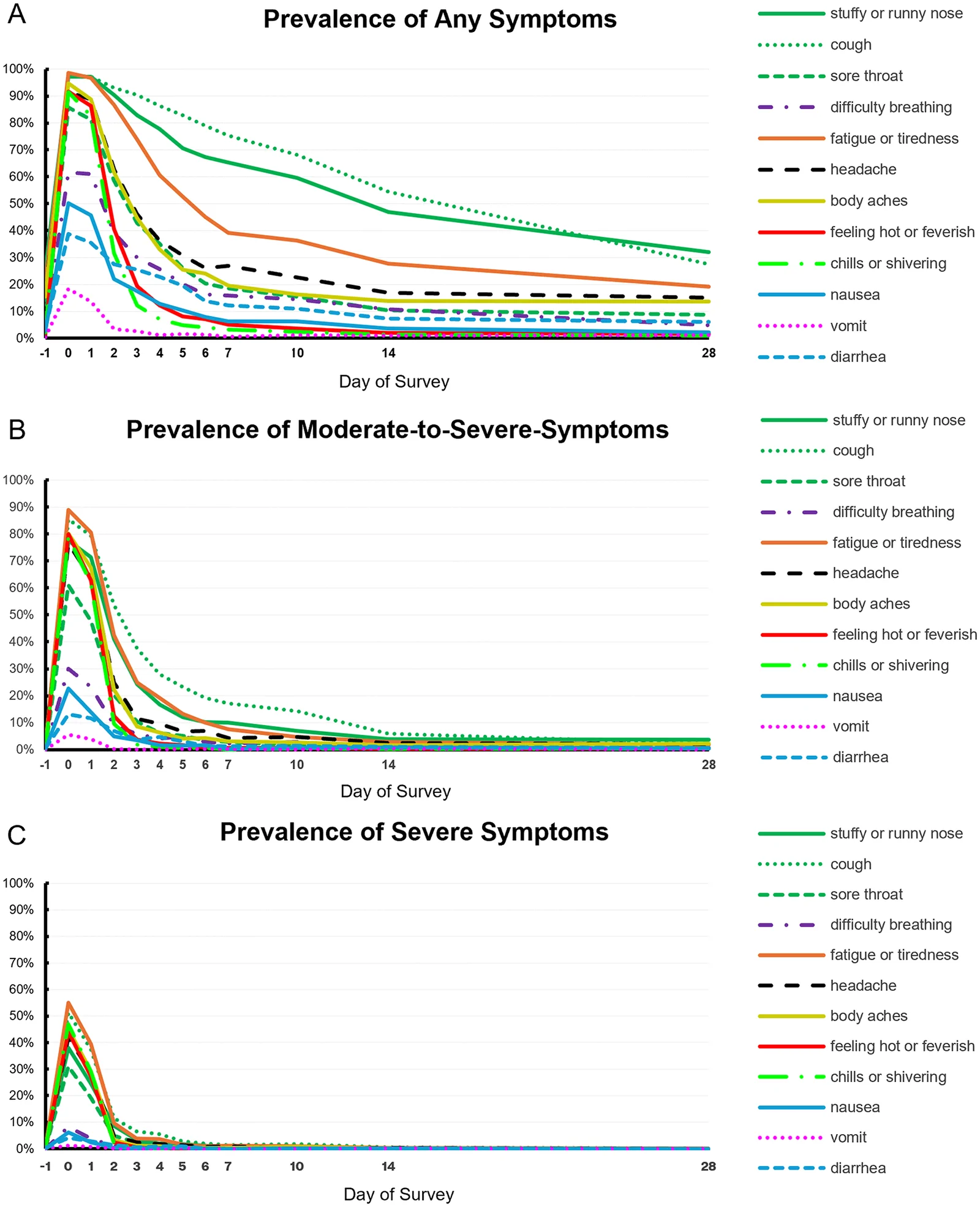
Prevalence of acute symptoms over 4 weeks: **A**) Any symptoms; **B**) Moderate to Severe; **C**) Severe. Day − 1 reflects the period prior to infection or symptom onset; Day 0 represents the worst moment of illness since infected as reported by participants

On Day 1, the most commonly reported symptoms (> 80%) were stuffy or runny nose, cough, fatigue, headache, body aches, feeling hot or feverish, chills or shivering, and sore throat (Table S2 and Fig. 3A). Stuffy or runny nose and cough remained most prevalent at Day 7 (65.3% and 75.3% respectively, and Day 14 (47.0% and 54.6%) and persisted through Week 4 (32.0% and 27.4%, respectively) (Table S2 and Fig. 3A). At Day 7, fatigue (39.2%), headaches (26.9%), body aches (19.6%), and sore throat (18.5%) were still common declining to 27.8%, 16.9%, 13.8% and 10.3% by Day 14, and 19.2%, 15.0%, 13.6%, and 8.6% at Week 4. Fever and chills declined sharply, occurring in 5.0% and 3.2% of participants respectively at Day 7, and 1.9% and 1.4% at Day 14, with only ≤ 1.6% reporting these symptoms at Week 4.

For moderate or severe symptoms, fatigue (80.5%), cough (79.3%), and stuffy or runny nose (71.3%) were the most common on Day 1. Prevalence dropped quickly, with 17.3% reported with moderate-to-severe cough and 7.7% with moderate-to-severe fatigue at Day 7, ≤ 6% at Day 14, and ≤ 3.8% of participants reporting any symptom as moderate-to-severe by Week 4 (Table S2 and Fig. 3B).

For severe symptoms, fatigue (39.4%) and cough (37.4%) were most frequent on Day 1 (Table S2 and Fig. 3C). Severe symptom prevalence declined sharply by Day 7 (≤ 1.4%) and approached zero (≤ 0.6%) by Day 14, with only isolated cases (< 0.2%) of cough or fatigue remaining at Week 4 (Table S2 and Fig. 3C).

### Findings from MMRM: Symptom changes from baseline over time

**Fig. 4:**
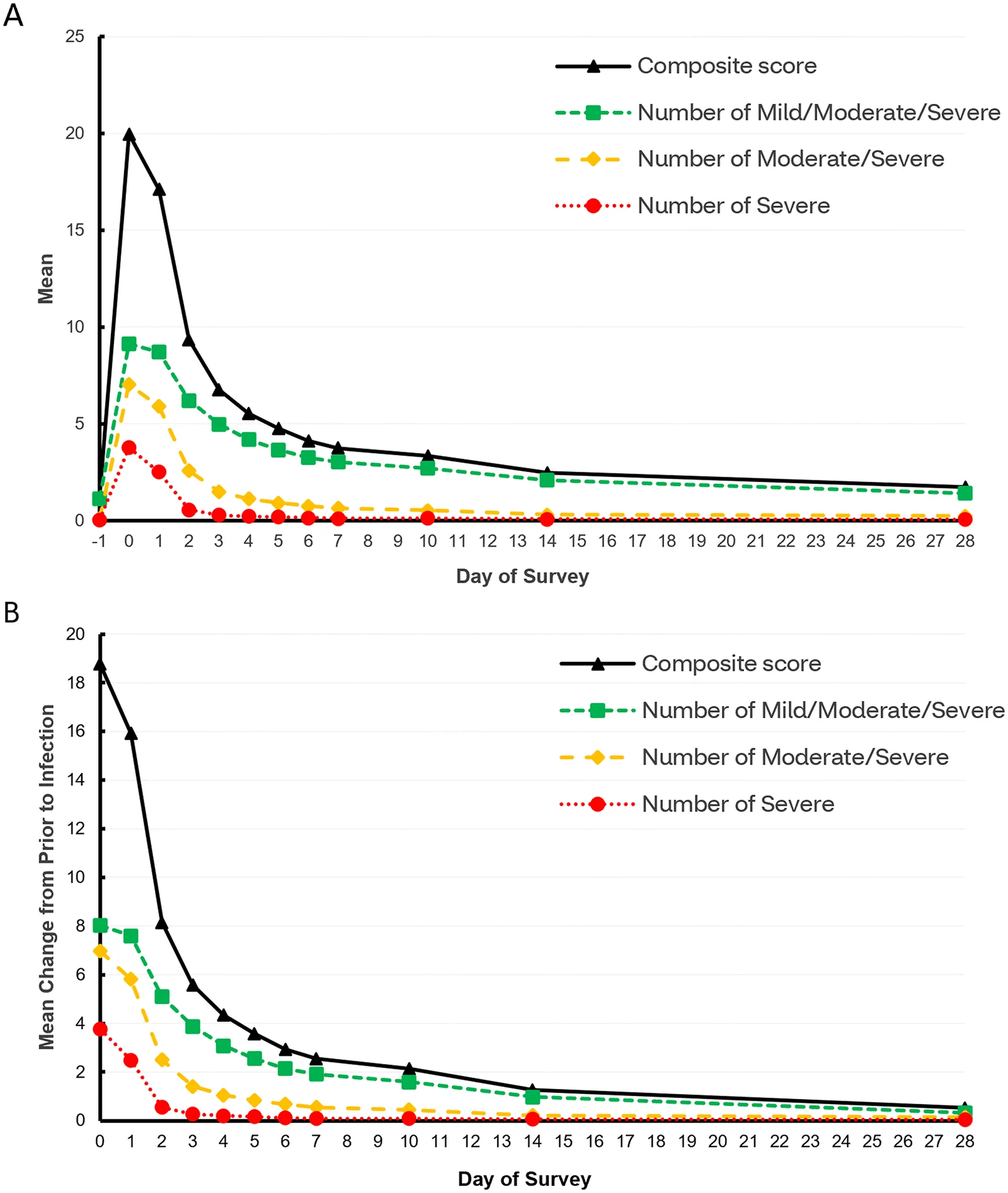
Symptom severity measures over 4 weeks (**A**) mean; (**B**) mean changes from prior to infection. Day − 1 reflects the period prior to infection or symptom onset; Day 0 represents the worst moment of illness since infected as reported by participants; Category Mild/Moderate/Severe refers to ‘Any reported symptoms’

The composite symptom score, number of any symptoms, and number of moderate-to-severe symptoms increased on Day 1 and remained elevated through Week 4. On Day 1, the mean composite symptom score was 17.1 (standard error, SE: 0.3); the mean number of any symptoms was 8.7 (SE: 0.1); and the mean number of moderate-to-severe symptoms was 5.9 (SE: 0.1) (Fig. 4A). All values were higher than baseline with p-values < 0.001 (Fig. 4B; Table 2). The symptom burden declined over time, though the number of any symptoms did persist through Week 4, with negligible reporting of severe symptoms after Day 5 (Table 2). Specifically, participants still reported higher mean composite symptom scores (+ 0.5; SE: 0.2), counts of any symptoms (+ 0.3; SE: 0.1), and moderate-to-severe symptoms (+ 0.2; SE: 0.1) at Week 4 compared to the pre-infection baseline (all *p* < 0.001). The number of severe symptoms increased on Day 1 (*p* < 0.001) and remained elevated through Day 5 (*p* = 0.039). On Day 1, participants experienced 2.5 severe symptoms (SE: 0.1) (Fig. 4A). The increase in the number of severe symptoms was higher than prior to infection through Day 5 [0.2 (SE: 0.1), *p* = 0.039]; there were no differences from Day 6 to Week 4 (all p-values > 0.05) (Fig. 4B; Table 2).

Across all symptom outcome measures, the greatest mean differences and largest effect sizes were observed at the worst moment since symptom onset followed by Day 1, with gradual improvement thereafter. For the composite symptom score, ESs were large on Days 1–7, medium on Days 10 and 14, and small at Week 4. ES for the mean number of any symptoms were large through Days 1–7, decreased to medium on Day 10 and small on Day 14 and Week 4. For moderate-to-severe symptoms, ES were large on Days 1–3, medium on Days 4–7, but small on Days 10 and 14, and Week 4. Though, for severe ratings, large effects were observed on Day 1, but small effects on Days 2–5. Observed scores and model parameter estimates are reported in Table S3 and S4 (Additional File 1).

### Findings from influenza high-risk group

#### Socio-demographic and clinical characteristics of high-risk group

A total of 225 participants (31.1%) met CDC criteria for high-risk for influenza complications [13], 188 of these participants were < 65 years. Demographics and clinical characteristics were broadly similar to the total sample, though mean age was higher (47.2 years), a greater proportion were vaccinated against influenza (54.2%), and antiviral use was common (85.8%) (Table 1). In addition, 352 participants were classified as high-risk for COVID-19 (Table S5, Additional File 1).

#### Prevalence of acute symptoms among adults at high risk for influenza

High-risk adults reported consistently higher symptom prevalence across most timepoints compared with the total population. For example, stuffy or runny nose was present in 30.7% of participants prior to infection, which increased to 97.8% on Day 1 and decreased to 39.4% at Week 4 (Table S6, Additional File 1), versus the total population who reported 25.1%, 97.2%, and 32.0%, at the same timepoints, respectively (Table S2). Similar patterns were observed for other symptoms and symptoms with moderate or severe levels and in both the < 65 years and COVID-19 high-risk groups (Table S7 and S8, Additional File 1).

#### Findings from MMRM: Symptom changes over time among the influenza high-risk group

Symptom trajectories in high-risk adults, including those < 65 and those at high-risk for COVID-19, closely mirrored the overall sample. Composite symptom score, the mean number of any symptoms, and the mean number of moderate-to-severe symptoms remained elevated through Week 4, while severe symptoms remained elevated only through Day 5 (*p* < 0.05) (Table 2 and Table S9, Additional File 1).

## Discussion

This study assessed the prevalence of individual symptoms, mean number of symptoms by severity and the mean composite symptom score during the first month of illness among symptomatic U.S. adults with influenza confirmed by rapid influenza diagnostic tests or rapid molecular assays for influenza infection. While symptom burden was highest in the initial days following symptom onset, the severity, prevalence, and mean number of symptoms declined substantially within 7–14 days, although some milder symptoms were still reported at Week 4. Time to resolution varied by symptom severity. Severe symptom ratings returned to pre-infection levels by Day 6, while mild to moderate symptoms remained present through Week 4, reflecting improvement from severe to milder symptom categories during the first week. These different severity trajectories provide complimentary insights into acute burden, functional impairments and longer-lasting residual symptoms.

In comparison to a large multi-season study of U.S. adults with acute respiratory illness that used an 8-symptom severity score (*N* = 2,374) [20], the sample in this study (*N* = 724), using a comparable set of eight symptoms, reported a higher mean symptom score (14.0 vs. 12.3). This is likely due to the younger age distribution (< 50 years: 70.9% vs. 55.0%), and the elevated burden during the 2024–2025 high-severity season. Despite these differences, the four most prevalent symptoms were consistent across studies: cough, nasal congestion, fatigue, and headache. Higher symptom prevalence in this study may reflect differences in patient characteristics, and the influenza season examined. The longitudinal design and broader symptom assessment provide new insights by capturing real-world trajectories in an outpatient cohort during a recent high-severity season.

The persistence of milder symptoms being present at 4 weeks’ follow-up highlights the extended burden of influenza illness among adults. This is consistent with clinical guidance noting that uncomplicated influenza usually resolves within about a week for most individuals [2, 33, 34], although certain symptoms like cough or fatigue may persist for more than two weeks.

Previous research using community-based samples often used clinical indicators such as admission to hospital and number of days of illness to assess severity of illness [35], which primarily reflect complications rather than day-to-day symptom experience and therefore may underestimate the true symptom burden. By incorporating patient perspectives, this research adds to the limited evidence base on patients’ assessments of influenza symptom severity [15, 20, 25], while also highlighting the prolonged symptom burden experienced by otherwise healthy adults and among high-risk groups. Findings should be interpreted in context and, as part of good science, future research is encouraged to examine and confirm (or not) the same set of issues studied here.

High-risk subgroup analyses showed broadly similar trajectories but higher prevalence across all timepoints, suggesting greater cumulative burden. Research in the U.S. has consistently shown that adults aged 65 years and older are disproportionately impacted by influenza, experiencing higher rates of influenza-related hospitalizations and deaths compared to younger age groups [8–11]. The increased risk has been attributed to increased likelihood of chronic conditions fueled by increasing age and the gradual worsening of the immune system due to aging [36]. Similarly, adults of any age with underlying health conditions are at increased risk of severe disease and complications from influenza infection [13]. These findings reinforce the importance of prioritizing high-risk groups in prevention and treatment strategies.

Several limitations should be considered when interpreting these findings. All exposures and outcomes were self-reported and therefore subject to recall error and potential misclassification. Key measures of pre-influenza (baseline) and peak (worst moment) symptom burden were based on retrospective recall at the Day 1 survey. Participants may not accurately remember their symptom levels and may be more likely to recall their most severe symptoms than their overall experience, which could lead to overestimation of peak symptoms and misclassification of baseline health status. Misclassification in survey-based studies is often nondifferential and would bias estimates towards the null (no association between self-reported exposures and outcome). However, prior research suggests that retrospective collection of patient-reported outcomes over short recall periods can demonstrate reasonable validity, including in U.S. COVID-19 populations using EQ-5D-5L measures [37]. Despite this, further research is needed to better understand the impact of recall bias on retrospective assessments of symptom severity and peak symptom burden.

Although all participants had test-confirmed influenza, the absence of a non-infected control group or comparator group with other respiratory infections limits the ability to determine whether observed symptom trajectories are specific to influenza or reflective of acute respiratory illness more broadly. However, the use of within-person comparisons to pre-infection baseline symptom levels partially mitigates this limitation by enabling evaluation of changes associated with the influenza episode. As such, these findings should be interpreted as descriptive of symptom burden among individuals with influenza rather than as influenza-specific relative to other respiratory infections.

Attrition was moderate: by Week 4, 224 of 724 participants (30.9%) had discontinued, leaving a retention rate of 69.1% (Table 2). Among those who dropped out, 121 responded to the attrition survey, with the most common reasons for participant discontinuation being lack of time or forgetting (61.2%), technical difficulties (13.2%), and feeling too unwell (11.6%) (Table S10, Additional File 1). Compared with participants who remained in the study, those who discontinued were more likely to be non-White, unvaccinated, and from communities with higher social vulnerability, and had a lower prevalence of endocrine disorders (Table S11, Additional File 1). These differences suggest possible selection bias, with relatively greater retention of vaccinated and health-conscious individuals, and underrepresentation of more vulnerable populations. The differential attrition could have led to an underestimation of symptom burden as vaccinated individuals typically experience less severe illness [38], and more health-conscious individuals tend to engage in healthier behaviors and may be less clinically vulnerable overall [39]. In addition to attrition over follow-up, the substantial proportion of individuals who did not engage or complete enrollment procedures raises the potential of nonresponse bias at study entry, as participants may differ systematically from those who did not participate. At the same time, individuals whose symptoms resolved early may have been less motivated to continue participating, which could result in overestimation of the Week 4 estimates. A sensitivity analysis comparing the full analytic cohort with participants who completed all scheduled surveys showed nearly identical mean changes from baseline in composite symptoms scores and mean number of severe symptoms, suggesting that differential survey completion had minimal impact on observed symptom trajectories. Although nonresponse bias cannot be fully excluded, this is consistent with evidence that lower response rates do not necessarily correspond to greater nonresponse bias [40, 41]. Furthermore, after examining participant characteristics across key assessment time points, participant demographics remained stable over time, suggesting that attrition did not materially affect cohort composition (Table S12, Additional File 1).

The analytic cohort was not fully representative of the broader U.S. population with influenza but reflects symptomatic adults seeking outpatient testing and care within a national ambulatory care clinic network. The analytic cohort (*n* = 724) was compared with the 50,003 individuals with influenza initially contacted but not included due to non-engagement, ineligibility, or incomplete enrollment procedures to help contextualize generalizability to individuals with influenza who sought care at these clinics. Participants were predominantly female (74.2% vs. 59.9%, *p* < 0.001) and White (76.2% vs. 62.7%, *p* < 0.001) and had lower mean SVI scores (0.32 vs. 0.35, *p* = 0.002) than excluded individuals (Table S13, Additional File 1). Given the over-representation of females and Whites, generalizability to male outpatients and non-White populations may be limited. However, a similar predominance of females and Whites has been observed in comparable survey-based research [29], reflecting a broader participation trend rather than a study-specific limitation. Additionally, the differences in SVI may affect generalizability, as populations with higher social vulnerability can differ in care-seeking behaviors, baseline health, and ability to participate in PRO surveys. Higher SVI is also associated with factors such as greater comorbidity burden, barriers to timely care, limited access to preventive and early treatment measures (e.g. influenza vaccination and outpatient antiviral therapy), and increased environmental stressors [42], which may influence the severity of illness. These differences may also affect observed symptom trajectories, as delayed care, differences in treatment access, and underlying health status may contribute to prolonged symptom duration and slower recovery over follow-up. Recruitment through the national ambulatory care clinic network may also reflect a healthier, more health-engaged population, given greater access to preventive care and antivirals (reported by 85.1% of participants) [43]. However, influenza vaccination coverage in this sample (49.3%) was similar to national adult estimates for the 2024–2025 season (44.6%), indicating broadly comparable preventive-care behavior [44]. Additionally, findings may not generalize to more clinically vulnerable groups typically captured in hospital-based or acute care settings. The prevalence of comorbidities in this cohort was lower than in hospitalized influenza populations, likely reflecting a less medically complex, community-based outpatient sample [21]. Participants were recruited via email and the study was limited to English-speaking participants, introducing potential selection bias against individuals without internet access in rural areas and may limit the socio-demographic representativeness of the sample.

Given the timing of participant enrollment, which was a median of three days from symptom onset, this may have led to underrepresentation of early symptom dynamics. Furthermore, this study used rapid antigen tests which generally have lower sensitivity than Polymerase Chain Reaction (PCR) laboratory tests for detecting influenza, hence there may be potential misclassification of influenza status.

Although the provision of participant compensation was designed to be ethically appropriate and proportionate to participant burden, this may have influenced retention and reporting behavior. The results are specific to the high-severity 2024–2025 influenza season, the first such season since 2017–2018 [21]. Patterns of symptom burden may differ in lower-severity seasons or with circulation of different influenza strains. As symptom ratings were based on participants’ subjective perception, some variability in individual reporting is expected. However, the composite symptom score demonstrated high internal consistency (Cronbach’s α = 0.88), supporting the reliability of this measure. Lastly, symptoms may not contribute equally to burden, perhaps a weighted scoring method may capture the burden more accurately.

Despite these limitations, this study has numerous strengths, including the use of patient-reported data to capture the subjective experience of influenza illness. These data provide a comprehensive view of symptom burden and recovery patterns revealing dimensions often missed through clinical indicators alone [45]. They also offer valuable insights into the real-world impact of influenza, specifically the prevalence, severity, and persistence of symptoms, key aspects during high-severity seasons. Such understanding can inform recovery expectations, return-to-work decisions, and guide clinical and public health responses.

A key strength of this study is its extended one-month follow-up, which enabled a more detailed assessment of symptom burden during influenza infection. A prior study of 317 adults tracked the frequency and severity of 34 symptoms over 14 days focusing on the feasibility of electronic symptom monitoring [19]. Building on that foundation, this study captured symptom prevalence, severity, and mean symptom count over a longer period. By prospectively collecting real-world patient-reported data from symptomatic outpatients with test-confirmed influenza illness across the U.S., this study provides a time-sensitive view of symptom burden over a one-month follow-up period. Participants were enrolled within 48 h of testing positive, minimizing, though not eliminating, recall bias, and allowing for the examination of variations from pre-infection in symptom prevalence, severity, and duration among both general and high-risk populations.

## Conclusion

Adults diagnosed with influenza during the 2024–2025 season experienced a high symptom burden. Severe symptom ratings returned to pre-infection levels by Day 6, while mild to moderate symptoms remained present through Week 4, reflecting improvement from severe to milder symptom categories during the first week. High-risk adults reported a higher prevalence of symptoms compared with the total population, although the duration of burden was similar. These findings emphasize the importance of longitudinal patient-reported symptom tracking to capture the full trajectory of influenza symptoms. This research highlights the ongoing burden of influenza symptoms on patients which may affect daily functioning and delay recovery. Importantly, the results support continued investment strategies to mitigate the burden of influenza, including vaccination, and reinforce the public-health relevance of monitoring outpatient symptom trajectories to inform clinical care, workplace policies, and seasonal preparedness. Future research should further explore the impact of vaccination on symptom burden and evaluate how influenza symptoms affect health-related quality of life and productivity, particularly in outpatient populations, to better understand the broader humanistic and societal burden of influenza.

## Supplementary Information

Below is the link to the electronic supplementary material.

Supplementary Material 1

## Data Availability

Aggregated data that support the findings of this study are available upon reasonable request from the corresponding author Tianyan Hu, subject to review. These data are not publicly available because they contain information that could compromise research participant privacy/consent.

## Abbreviations

BMI: Body mass index
CDC: Centers for Disease Control and Prevention
COVID-19: Coronavirus disease 2019
ES: Effect size
LSE: Least squares estimates
MMRM: Mixed models for repeated measures
SD: Standard deviation
SE: Standard error
STROBE: Strengthening the Reporting of Observational studies in Epidemiology
SVI: Social Vulnerability Index
US: United States
