# Supplementary material for: The one-month impact of influenza infection on patient-reported symptom burden among symptomatic U.S. adults with a test-confirmed illness: a longitudinal study
Source: BMC Infect Dis. 2026 Jul 9;26:1754. doi: 10.1186/s12879-026-13950-y (PMC13632369; doi:10.1186/s12879-026-13950-y)
Supplement: Supplementary file 1 [file 12879_2026_13950_MOESM1_ESM.docx]

# ADDITIONAL FILE 1

**Complete list of health conditions assessed during first survey**

During the first survey on Day 1 (enrollment day), participants were asked whether they received a diagnosis from a healthcare provider for any of the following comorbidities listed in the table below or are currently pregnant.

**Table S1.** List of underlying health conditions associated with increased risk for flu or COVID-19 complications

| **Disease/Condition** | **High risk for influenza complications** | **High risk for COVID-19 complications** |
| --- | --- | --- |
| Any current/active cancers or malignancies (other than skin cancer) | ✓ | ✓ |
| Cerebrovascular disease (such as stroke, transient ischemic attack [TIA]) | ✓ | ✓ |
| Chronic kidney disease (reduced kidney function or on dialysis) | ✓ | ✓ |
| Chronic lung conditions (such as asthma, chronic obstructive pulmonary disease [COPD, emphysema], bronchiectasis, cystic fibrosis, pulmonary embolism [blood clots in lungs], pulmonary fibrosis, interstitial lung disease [ILD]) | ✓ | ✓ |
| Chronic liver disease (such as cirrhosis, fatty liver, alcoholic liver disease, autoimmune hepatitis) | ✓ | ✓ |
| Endocrine disorders (such as diabetes [type 1 or type 2]) | ✓ | ✓ |
| Heart conditions (such as heart attack, heart failure, coronary artery disease, bypass surgery/stent, cardiomyopathy) | ✓ | ✓ |
| Mental health conditions (such as mood disorders, depression, schizophrenia) | X | ✓ |
| Obesity: BMI>30 | X | ✓ |
| Obesity: BMI>40 | ✓ | X |
| Weakened immune system/ immunocompromised (currently on chemotherapy, chronic corticosteroids [such as prednisone], or anti-rejection medication due to an organ transplant; genetic immune disorders; other immune disorders, such as HIV or primary immunodeficiencies) | ✓ | ✓ |
| Blood disorders (such as sickle cell disease) | ✓ | X |
| Current or former smoker | X | ✓ |
| Active tuberculosis | ✓ | ✓ |
| Pregnant | ✓ | ✓ |

COVID-19=coronavirus disease 2019; COPD=chronic obstructive lung disease; BMI=body mass index; HIV=human immunodeficiency virus; ILD= interstitial lung disease; TIA=transient ischemic attack.

**Table S2**. Prevalence of self-reported acute symptoms by severity from pre-infection to follow-up over a 1-month period among influenza-infected adults in total population

| **Acute symptoms** | **Time of Survey** | | | | | | | | | | | |
| --- | --- | --- | --- | --- | --- | --- | --- | --- | --- | --- | --- | --- |
|  | **Prior to infection**  **(N=724)** | **Worst moment**  **(N=724)** | **Day 1**  **(N=724)** | **Day 2**  **(N=607)** | **Day 3 (N=602)** | **Day 4**  **(N=586)** | **Day 5**  **(N=575)** | **Day 6**  **(N=565)** | **Day 7**  **(N=562)** | **Day 10**  **(N=522)** | **Day 14**  **(N=515)** | **Week 4**  **(N=500)** |
| **Any symptoms** |  |  |  |  |  |  |  |  |  |  |  |  |
| stuffy or runny nose | 25.1 | 97.2 | 97.2 | 90.4 | 82.9 | 77.8 | 70.6 | 67.3 | 65.3 | 59.6 | 47.0 | 32.0 |
| cough | 14.2 | 97.7 | 97.2 | 93.1 | 90.4 | 86.4 | 83.0 | 78.9 | 75.3 | 68.2 | 54.6 | 27.4 |
| fatigue or tiredness | 19.9 | 98.6 | 96.8 | 86.7 | 73.9 | 60.6 | 52.7 | 45.1 | 39.2 | 36.2 | 27.8 | 19.2 |
| headache | 16.9 | 91.4 | 88.8 | 62.9 | 46.0 | 36.4 | 30.8 | 26.0 | 26.9 | 22.6 | 16.9 | 15.0 |
| body aches | 15.3 | 94.8 | 88.8 | 61.6 | 44.7 | 32.9 | 25.4 | 24.1 | 19.6 | 16.3 | 13.8 | 13.6 |
| feeling hot or feverish | 1.4 | 91.7 | 86.3 | 40.5 | 19.3 | 12.1 | 8.2 | 7.1 | 5.0 | 3.6 | 1.9 | 1.6 |
| chills or shivering | 2.1 | 91.4 | 82.7 | 31.6 | 12.1 | 7.0 | 4.9 | 4.1 | 3.2 | 2.3 | 1.4 | 1.0 |
| sore throat | 6.6 | 85.8 | 81.2 | 58.7 | 42.9 | 35.2 | 26.1 | 20.4 | 18.5 | 15.5 | 10.3 | 8.6 |
| difficulty breathing | 3.7 | 61.6 | 61.1 | 39.0 | 29.9 | 25.6 | 20.2 | 16.3 | 15.8 | 14.4 | 10.7 | 4.8 |
| nausea | 2.8 | 50.1 | 45.7 | 21.9 | 17.4 | 12.8 | 10.3 | 8.0 | 6.2 | 6.1 | 3.5 | 2.2 |
| vomit | 0.3 | 18.4 | 13.7 | 3.3 | 2.5 | 1.0 | 1.6 | 1.4 | 0.5 | 1.2 | 1.0 | 1.2 |
| diarrhea | 3.9 | 38.8 | 35.4 | 27.5 | 25.4 | 22.9 | 19.5 | 13.8 | 12.1 | 10.9 | 7.2 | 6.0 |
| **Moderate-to-severe** |  |  |  |  |  |  |  |  |  |  |  |  |
| stuffy or runny nose | 1.5 | 77.5 | 71.3 | 41.0 | 24.3 | 16.7 | 12.0 | 10.3 | 10.0 | 6.9 | 3.9 | 3.8 |
| cough | 1.1 | 85.5 | 79.3 | 53.5 | 37.5 | 28.0 | 23.3 | 19.1 | 17.3 | 14.4 | 6.0 | 2.2 |
| fatigue or tiredness | 1.0 | 89.0 | 80.5 | 42.5 | 24.9 | 19.1 | 13.2 | 10.1 | 7.7 | 4.8 | 2.9 | 1.8 |
| headache | 2.2 | 76.5 | 63.7 | 24.9 | 11.5 | 9.6 | 6.8 | 7.1 | 4.3 | 4.8 | 2.5 | 2.2 |
| body aches | 0.6 | 80.3 | 67.5 | 22.2 | 8.6 | 6.3 | 4.2 | 4.3 | 3.2 | 2.7 | 1.8 | 2.2 |
| feeling hot or feverish | 0.1 | 80.0 | 62.9 | 12.5 | 3.7 | 2.2 | 1.7 | 0.9 | 0.7 | 0.6 | 0.2 | 0.4 |
| chills or shivering | 0.0 | 78.7 | 61.7 | 9.6 | 1.8 | 1.0 | 0.7 | 0.7 | 0.0 | 0.4 | 0.2 | 0.4 |
| sore throat | 0.3 | 61.1 | 47.8 | 20.3 | 10.5 | 6.1 | 5.2 | 3.5 | 2.9 | 1.7 | 0.6 | 0.6 |
| difficulty breathing | 0.1 | 30.1 | 23.3 | 9.2 | 5.8 | 4.1 | 4.9 | 2.8 | 2.3 | 2.9 | 1.0 | 0.6 |
| nausea | 0.1 | 22.7 | 13.8 | 4.9 | 3.5 | 1.7 | 1.4 | 1.6 | 0.2 | 0.6 | 0.8 | 0.4 |
| vomit | 0.1 | 5.7 | 4.0 | 0.2 | 0.0 | 0.0 | 0.4 | 0.2 | 0.2 | 0.0 | 0.4 | 0.0 |
| diarrhea | 0.4 | 13.1 | 11.7 | 6.9 | 3.8 | 4.8 | 3.1 | 1.4 | 1.3 | 1.5 | 1.2 | 0.6 |
| **Severe** |  |  |  |  |  |  |  |  |  |  |  |  |
| stuffy or runny nose | 0.1 | 38.0 | 24.6 | 8.6 | 3.5 | 2.1 | 1.7 | 1.1 | 0.4 | 0.8 | 0.0 | 0.0 |
| cough | 0.1 | 51.4 | 37.4 | 11.5 | 6.5 | 5.5 | 2.8 | 2.0 | 1.4 | 1.7 | 0.6 | 0.2 |
| fatigue or tiredness | 0.1 | 55.0 | 39.4 | 9.6 | 3.8 | 3.6 | 1.6 | 0.9 | 1.1 | 1.0 | 0.4 | 0.2 |
| headache | 0.0 | 42.0 | 28.6 | 4.9 | 2.5 | 1.7 | 1.4 | 0.7 | 0.9 | 0.2 | 0.4 | 0.0 |
| body aches | 0.0 | 45.2 | 29.0 | 3.1 | 1.2 | 0.9 | 0.4 | 0.4 | 0.4 | 0.8 | 0.2 | 0.0 |
| feeling hot or feverish | 0.1 | 44.1 | 27.2 | 2.3 | 0.5 | 0.2 | 0.2 | 0.2 | 0.0 | 0.2 | 0.0 | 0.0 |
| chills or shivering | 0.0 | 47.0 | 29.0 | 2.0 | 0.3 | 0.2 | 0.4 | 0.2 | 0.0 | 0.2 | 0.0 | 0.0 |
| sore throat | 0.1 | 31.1 | 19.2 | 4.8 | 2.5 | 1.5 | 1.2 | 0.9 | 0.4 | 0.2 | 0.0 | 0.0 |
| difficulty breathing | 0.0 | 8.3 | 3.9 | 1.0 | 0.8 | 0.7 | 0.9 | 0.7 | 0.2 | 0.2 | 0.0 | 0.0 |
| nausea | 0.0 | 6.1 | 2.4 | 0.3 | 0.2 | 0.3 | 0.4 | 0.0 | 0.0 | 0.2 | 0.2 | 0.0 |
| vomit | 0.1 | 1.2 | 0.8 | 0.2 | 0.0 | 0.0 | 0.4 | 0.0 | 0.0 | 0.0 | 0.2 | 0.0 |
| diarrhea | 0.3 | 4.0 | 2.8 | 1.3 | 0.5 | 0.9 | 0.9 | 0.2 | 0.0 | 0.0 | 0.0 | 0.0 |

**Table S3**. Summary of observed mean symptom outcome scores, score change, and effect size from pre-infection to follow-up over a 1-month period among flu-infected adults in total population.

|  | **Total population** | | | | **Influenza high-risk group (all ages)** | | | |
| --- | --- | --- | --- | --- | --- | --- | --- | --- |
|  | **n** | **Score**  **Mean (SD)** | **Score change**  **Mean (SD)** | **ES** | **n** | **Score**  **Mean (SD)** | **Score change**  **Mean (SD)** | **ES** |
| **Composite acute symptom score** | | | | | | | | |
| Prior to infection | 724 | 1.2 (1.6) |  |  | 225 | 1.5 (1.8) |  |  |
| Worst Moment | 724 | 19.9 (5.8) | 18.7 (5.8) | 3.23 | 225 | 19.9 (6.1) | 18.4 (6.1) | 3.03 |
| Day 1 | 724 | 17.1 (6.1) | 15.9 (6.1) | 2.59 | 225 | 17.0 (6.4) | 15.5 (6.4) | 2.43 |
| Day 2 | 607 | 9.1 (5.2) | 7.9 (5.2) | 1.54 | 192 | 9.8 (5.5) | 8.2 (5.6) | 1.48 |
| Day 3 | 602 | 6.5 (4.4) | 5.3 (4.3) | 1.22 | 191 | 7.0 (4.6) | 5.5 (4.6) | 1.18 |
| Day 4 | 586 | 5.3 (4.0) | 4.1 (3.9) | 1.05 | 186 | 6.1 (4.2) | 4.6 (4.3) | 1.07 |
| Day 5 | 575 | 4.4 (3.9) | 3.2 (3.9) | 0.84 | 184 | 5.3 (4.5) | 3.9 (4.6) | 0.84 |
| Day 6 | 565 | 3.8 (3.5) | 2.6 (3.5) | 0.74 | 180 | 4.5 (3.9) | 3.0 (4.1) | 0.73 |
| Day 7 | 562 | 3.4 (3.1) | 2.2 (3.1) | 0.72 | 181 | 4.3 (3.4) | 2.8 (3.5) | 0.80 |
| Day 10 | 522 | 3.0 (3.0) | 1.8 (3.1) | 0.59 | 173 | 3.7 (3.1) | 2.3 (3.1) | 0.73 |
| Day 14 | 515 | 2.2 (2.4) | 1.0 (2.5) | 0.41 | 171 | 2.8 (2.6) | 1.3 (2.6) | 0.50 |
| Week 4 | 500 | 1.5 (2.0) | 0.3 (2.2) | 0.14 | 165 | 1.9 (2.3) | 0.4 (2.5) | 0.18 |
| **Number of any symptoms** | | | | | | | | |
| Prior to infection | 724 | 1.1 (1.4) |  |  | 225 | 1.4 (1.6) |  |  |
| Worst Moment | 724 | 9.2 (1.7) | 8.1 (2.0) | 3.97 | 225 | 9.2 (1.8) | 7.7 (2.2) | 3.56 |
| Day 1 | 724 | 8.8 (2.0) | 7.6 (2.3) | 3.33 | 225 | 8.7 (2.2) | 7.3 (2.4) | 3.03 |
| Day 2 | 607 | 6.2 (2.5) | 5.1 (2.6) | 1.93 | 192 | 6.5 (2.6) | 5.1 (2.8) | 1.80 |
| Day 3 | 602 | 4.9 (2.4) | 3.8 (2.5) | 1.48 | 191 | 5.2 (2.5) | 3.7 (2.7) | 1.38 |
| Day 4 | 586 | 4.1 (2.3) | 3.0 (2.4) | 1.25 | 186 | 4.6 (2.4) | 3.2 (2.6) | 1.23 |
| Day 5 | 575 | 3.5 (2.3) | 2.4 (2.4) | 1.03 | 184 | 4.1 (2.5) | 2.7 (2.7) | 1.01 |
| Day 6 | 565 | 3.1 (2.2) | 2.0 (2.3) | 0.86 | 180 | 3.6 (2.4) | 2.2 (2.6) | 0.82 |
| Day 7 | 562 | 2.9 (2.2) | 1.8 (2.3) | 0.78 | 181 | 3.5 (2.3) | 2.1 (2.5) | 0.83 |
| Day 10 | 522 | 2.6 (2.1) | 1.5 (2.3) | 0.66 | 173 | 3.1 (2.2) | 1.7 (2.3) | 0.73 |
| Day 14 | 515 | 2.0 (1.9) | 0.9 (2.0) | 0.44 | 171 | 2.5 (1.9) | 1.1 (2.1) | 0.52 |
| Week 4 | 500 | 1.3 (1.7) | 0.2 (1.8) | 0.14 | 165 | 1.7 (1.8) | 0.4 (2.0) | 0.19 |
| **Number of moderate to severe acute symptoms** | | | | | | | | |
| Prior to infection | 724 | 0.1 (0.3) |  |  | 225 | 0.1 (0.3) |  |  |
| Worst Moment | 724 | 7.0 (2.3) | 6.9 (2.4) | 2.94 | 225 | 7.0 (2.5) | 6.9 (2.5) | 2.76 |
| Day 1 | 724 | 5.9 (2.7) | 5.8 (2.7) | 2.16 | 225 | 5.8 (2.8) | 5.7 (2.8) | 2.01 |
| Day 2 | 607 | 2.5 (2.3) | 2.4 (2.3) | 1.02 | 192 | 2.7 (2.4) | 2.6 (2.4) | 1.08 |
| Day 3 | 602 | 1.4 (1.8) | 1.3 (1.8) | 0.71 | 191 | 1.5 (1.8) | 1.4 (1.8) | 0.78 |
| Day 4 | 586 | 1.0 (1.6) | 0.9 (1.6) | 0.59 | 186 | 1.3 (1.8) | 1.2 (1.8) | 0.68 |
| Day 5 | 575 | 0.8 (1.5) | 0.7 (1.5) | 0.46 | 184 | 1.0 (1.7) | 1.0 (1.7) | 0.56 |
| Day 6 | 565 | 0.6 (1.3) | 0.5 (1.3) | 0.40 | 180 | 0.8 (1.6) | 0.7 (1.6) | 0.46 |
| Day 7 | 562 | 0.5 (1.0) | 0.4 (1.0) | 0.40 | 181 | 0.7 (1.2) | 0.6 (1.2) | 0.53 |
| Day 10 | 522 | 0.4 (1.0) | 0.3 (1.0) | 0.32 | 173 | 0.6 (1.0) | 0.5 (1.0) | 0.48 |
| Day 14 | 515 | 0.2 (0.7) | 0.1 (0.8) | 0.18 | 171 | 0.3 (0.8) | 0.2 (0.8) | 0.25 |
| Week 4 | 500 | 0.2 (0.5) | 0.1 (0.6) | 0.12 | 165 | 0.2 (0.7) | 0.1 (0.7) | 0.12 |
| **Number of severe acute symptoms** | | | | | | | | |
| Prior to infection | 724 | 0.0 (0.1) |  |  | 225 | 0.0 (0.1) |  |  |
| Worst Moment | 724 | 3.7 (2.6) | 3.7 (2.6) | 1.43 | 225 | 3.7 (2.7) | 3.7 (2.7) | 1.39 |
| Day 1 | 724 | 2.4 (2.3) | 2.4 (2.3) | 1.06 | 195 | 2.5 (2.4) | 2.5 (2.4) | 1.05 |
| Day 2 | 611 | 0.5 (1.2) | 0.5 (1.2) | 0.42 | 192 | 0.6 (1.4) | 0.6 (1.4) | 0.40 |
| Day 3 | 604 | 0.2 (0.7) | 0.2 (0.7) | 0.29 | 186 | 0.3 (0.8) | 0.3 (0.8) | 0.34 |
| Day 4 | 586 | 0.2 (0.7) | 0.2 (0.7) | 0.24 | 184 | 0.2 (0.6) | 0.2 (0.6) | 0.31 |
| Day 5 | 576 | 0.1 (0.7) | 0.1 (0.7) | 0.15 | 180 | 0.2 (1.0) | 0.2 (1.0) | 0.19 |
| Day 6 | 565 | 0.1 (0.4) | 0.1 (0.4) | 0.14 | 181 | 0.1 (0.4) | 0.1 (0.4) | 0.19 |
| Day 7 | 562 | 0.0 (0.3) | 0.0 (0.3) | 0.10 | 173 | 0.1 (0.4) | 0.0 (0.4) | 0.13 |
| Day 10 | 522 | 0.1 (0.3) | 0.0 (0.4) | 0.11 | 171 | 0.1 (0.4) | 0.1 (0.4) | 0.14 |
| Day 14 | 515 | 0.0 (0.2) | 0.0 (0.2) | 0.03 | 165 | 0.0 (0.2) | 0.0 (0.2) | 0.11 |
| Week 4 | 500 | 0.0 (0.1) | 0.0 (0.1) | -0.07 | 225 | 0.0 (0.0) | 0.0 (0.1) | -0.11 |

SD=standard deviation; ES=effect size. The within-cohort effect size (ES) (Cohen’s d) was determined by estimating the mean change from prior to infection to follow-up, divided by the SD of change scores. These standardized effect sizes were interpreted using Cohen’s conventions, with threshold values of 0.2 to <0.5, 0.5 to <0.8, and ≥0.8 indicating “small,” “medium,” and “large” effects, respectively.

**Table S4**. Parameter estimates for mixed-models for repeated measures for symptom outcomes (total population)

|  | **Composite acute symptom score** | **Number of acute symptoms** | **Number of moderate/ severe symptoms** | **Number of severe symptoms** |
| --- | --- | --- | --- | --- |
| Intercept | 20.33 (0.41) | 9.03 (0.18) | 7.18 (0.15) | 4.03 (0.12) |
| High Risk | -0.15 (0.46) | -0.14 (0.13) | 0.00 (0.19) | 0.04 (0.10) |
| **Time** |  |  |  |  |
| Worst moment | Reference | Reference | Reference | Reference |
| Day 1 | -2.66 (0.29) | -0.36 (0.09) | -0.96 (0.13) | -1.35 (0.09) |
| Day 2 | -11.06 (0.33) | -3.04 (0.14) | -4.53 (0.15) | -3.48 (0.09) |
| Day 3 | -13.72 (0.33) | -4.22 (0.14) | -5.73 (0.14) | -3.79 (0.10) |
| Day 4 | -15.32 (0.32) | -5.26 (0.14) | -6.22 (0.14) | -3.85 (0.10) |
| Day 5 | -16.13 (0.34) | -5.81 (0.14) | -6.41 (0.14) | -3.94 (0.10) |
| Day 6 | -16.75 (0.33) | -6.25 (0.14) | -6.55 (0.14) | -3.97 (0.10) |
| Day 7 | -17.31 (0.31) | -6.60 (0.14) | -6.73 (0.13) | -4.00 (0.10) |
| Day 10 | -17.62 (0.33) | -6.84 (0.15) | -6.81 (0.14) | -4.00 (0.10) |
| Day 14 | -18.54 (0.32) | -7.49 (0.14) | -7.02 (0.13) | -4.05 (0.10) |
| Week 4 | -19.19 (0.33) | -8.10 (0.13) | -7.06 (0.13) | -4.05 (0.10) |
| **High Risk by Time** |  |  |  |  |
| Worst moment | Reference | Reference | Reference | Reference |
| Day 1 | 0.00 (0.41) | 0.03 (0.13) | -0.11 (0.19) | 0.08 (0.13) |
| Day 2 | 0.94 (0.46) | 0.45 (0.19) | 0.37 (0.22) | 0.12 (0.13) |
| Day 3 | 1.00 (0.46) | 0.59 (0.19) | 0.32 (0.20) | 0.11 (0.13) |
| Day 4 | 1.17 (0.46) | 0.73 (0.19) | 0.42 (0.20) | 0.05 (0.13) |
| Day 5 | 1.25 (0.47) | 0.77 (0.20) | 0.38 (0.20) | 0.16 (0.14) |
| Day 6 | 1.07 (0.46) | 0.75 (0.20) | 0.33 (0.20) | 0.05 (0.14) |
| Day 7 | 1.18 (0.44) | 0.86 (0.19) | 0.33 (0.19) | 0.03 (0.14) |
| Day 10 | 0.93 (0.46) | 0.72 (0.20) | 0.22 (0.19) | 0.03 (0.14) |
| Day 14 | 0.80 (0.45) | 0.70 (0.19) | 0.10 (0.19) | 0.03 (0.14) |
| Week 4 | 0.56 (0.46) | 0.56 (0.19) | 0.04 (0.19) | 0.01 (0.14) |
| **Receipt of influenza vaccine by Time** |  |  |  |  |
| Worst moment | -1.51 (0.42) | -0.37 (0.12) | -0.47 (0.17) | -0.70 (0.10) |
| Day 1 | -1.87 (0.45) | -0.52 (0.15) | -0.74 (0.20) | -0.64 (0.10) |
| Day 2 | -1.55 (0.39) | -0.58 (0.19) | -0.70 (0.18) | -0.29 (0.10) |
| Day 3 | -1.45 (0.33) | -0.81 (0.18) | -0.43 (0.14) | -0.22 (0.11) |
| Day 4 | -0.89 (0.30) | -0.48 (0.18) | -0.28 (0.12) | -0.16 (0.11) |
| Day 5 | -0.90 (0.30) | -0.47 (0.18) | -0.28 (0.12) | -0.17 (0.11) |
| Day 6 | -0.77 (0.28) | -0.39 (0.18) | -0.28 (0.11) | -0.12 (0.11) |
| Day 7 | -0.51 (0.24) | -0.27 (0.17) | -0.17 (0.08) | -0.10 (0.11) |
| Day 10 | -0.46 (0.24) | -0.28 (0.17) | -0.12 (0.08) | -0.08 (0.11) |
| Day 14 | -0.25 (0.20) | -0.19 (0.15) | -0.03 (0.06) | -0.06 (0.11) |
| Week 4 | -0.19 (0.18) | -0.15 (0.14) | -0.02 (0.05) | -0.06 (0.11) |
| Baseline score | 0.40 (0.05) | 0.27 (0.03) | 0.23 (0.06) | -0.06 (0.22) |
| **Sex** |  |  |  |  |
| Female | Reference | Reference | Reference | Reference |
| Male | -0.27 (0.18) | -0.22 (0.11) | -0.02 (0.05) | -0.31 (0.07) |
| **Race/Ethnicity** |  |  |  |  |
| White | Reference | Reference | Reference | Reference |
| Black or African American | -0.16 (0.37) | 0.00 (0.22) | -0.11 (0.10) | -0.09 (0.14) |
| Hispanic or Latino | 0.44 (0.33) | -0.08 (0.19) | 0.15 (0.09) | 0.37 (0.12) |
| Asian | -0.25 (0.45) | -0.30 (0.27) | -0.14 (0.13) | -0.18 (0.17) |
| Other* | 0.17 (0.42) | 0.33 (0.25) | -0.05 (0.12) | 0.31 (0.16) |
| **US region** |  |  |  |  |
| Northeast | Reference | Reference | Reference | Reference |
| South | -0.23 (0.23) | -0.07 (0.14) | -0.05 (0.07) | 0.03 (0.09) |
| Midwest | -0.50 (0.22) | -0.26 (0.14) | -0.08 (0.06) | 0.02 (0.08) |
| West | -0.81 (0.39) | -0.52 (0.24) | -0.16 (0.11) | -0.08 (0.15) |
| Other/Unknown | -1.46 (1.18) | -0.15 (0.68) | -0.37 (0.34) | -0.21 (0.41) |
| **Social Vulnerability Index** |  |  |  |  |
| <0.25 | Reference | Reference | Reference | Reference |
| ≥0.25 and <0.5 | 0.13 (0.17) | 0.06 (0.10) | 0.07 (0.05) | 0.01 (0.06) |
| ≥0.5 and <0.75 | 0.03 (0.26) | -0.04 (0.16) | 0.09 (0.07) | -0.05 (0.10) |
| ≥0.75 | 0.82 (0.47) | 0.33 (0.28) | 0.39 (0.13) | 0.31 (0.17) |
| Antiviral use | 0.22 (0.22) | 0.30 (0.13) | 0.05 (0.06) | 0.08 (0.08) |

*American Indian or Alaska Native, Native Hawaiian or Other Pacific Islander, Multi-race, Prefer not to answer.

Note: Coefficients from categorical variables represent the adjusted mean difference in each outcome relative to the reference category. Positive values indicate higher burden, and negative values indicate lower burden. Standard errors are shown in parentheses. For continuous variables, each one-unit increase in the covariate X corresponds to β-unit change in the outcome Y.

**Table S5.** Baseline demographic and clinical characteristics of study participants in high-risk subgroups

|  | **Influenza high-risk group <65 years (N=188)** | **COVID-19 high-risk group: All ages (N=352)** |
| --- | --- | --- |
| **Age, years, mean (SD)** | 42.9 (11.8) | 44.1 (14.4) |
| **Sex, n (%)** |  |  |
| Female | 142 (75.5) | 268 (76.1) |
| Male | 46 (24.5) | 84 (23.9) |
| **Race/Ethnicity, n (%)** |  |  |
| White | 154 (81.9) | 294 (83.5) |
| Black or African American | 9 (4.8) | 16 (4.5) |
| Hispanic or Latino | 15 (8.0) | 24 (6.8) |
| Asian | 3 (1.6) | 5 (1.4) |
| Other^a^ | 7 (3.7) | 13 (3.7) |
| **US Region, n (%)** |  |  |
| Northeast | 34 (18.1) | 60 (17.0) |
| South | 83 (44.1) | 165 (46.9) |
| Midwest | 61 (32.4) | 108 (30.7) |
| West | 9 (4.8) | 18 (5.1) |
| Other\Unknown | 1 (0.5) | 1 (0.3) |
| **Employment status, n (%)** |  |  |
| Yes | 157 (83.5) | 283 (80.4) |
| **Social Vulnerability Index (SVI) ^b^, mean (SD)** | 0.34 (0.19) | 0.34 (0.19) |
| **SVI category, n (%)** |  |  |
| <0.25 | 73 (38.8) | 140 (39.8) |
| ≥0.25 and <0.5 | 82 (43.6) | 153 (43.5) |
| ≥0.5 and <0.75 | 24 (12.8) | 43 (12.2) |
| ≥0.75 | 8 (4.3) | 15 (4.3) |
| Anti-viral use, n (%) | 164 (87.2) | 299 (84.9) |
| Received the 2024-2025 influenza vaccine, n (%) | 93 (49.5) | 194 (55.1) |
| **Pregnant** |  |  |
| No | 130 (69.1) | 240 (68.2) |
| Yes | 9 (4.8) | 9 (2.6) |
| Not applicable | 49 (26.1) | 103 (29.3) |
| **Underlying health conditions, n (%)** |  |  |
| Any current/active cancers or malignancies (other than skin cancer) | 5 (2.7) | 5 (1.4) |
| Cerebrovascular disease | 5 (2.7) | 8 (2.3) |
| Chronic kidney disease | 3 (1.6) | 3 (0.9) |
| Chronic lung conditions | 45 (23.9) | 51 (14.5) |
| Chronic liver disease | 10 (5.3) | 11 (3.1) |
| Endocrine disorders (such as diabetes [type 1 or type 2]) | 28 (14.9) | 33 (9.4) |
| Heart conditions | 17 (9.0) | 21 (6.0) |
| Obesity: BMI>40 | 92 (48.9) | 104 (29.5) |
| Weakened immune system/ immunocompromised | 16 (8.5) | 17 (4.8) |
| Blood disorders ^b^ | 5 (2.7) | 2 (0.6) |
| Active Tuberculosis | 0 | 0 |
| Mental health conditions | - | 182 (51.7) |
| Smoker | - | 75 (21.3) |
| Number of comorbidities, mean (SD) | 1.20 (0.61)^d^ | 0.72 (0.78)^e^ |
| ≥1 comorbidity, n (%) | 180 (95.7)^d^ | 197 (56.0)^e^ |
| ≥1 comorbidity or pregnant, n (%) | 188 (100.0)^d^ | 205 (58.2)^e^ |

COVID-19=coronavirus disease 2019; SD=standard deviation; BMI=body mass index.

^a^American Indian or Alaska Native, Native Hawaiian or Other Pacific Islander, Patient Refused, Other, Unknown.

^b^ SVI is a score that ranges from 0 to 1. Higher values correspond to higher vulnerability

^c^ Blood disorders are considered high-risk for influenza but not for COVID-19. These 2 participants with blood disorders were included in the COVID-19 high risk subgroup based on other qualifying conditions.

^d^ High-risk criteria for influenza.

^e^ High-risk criteria for COVID-19.

**Table S6**. Prevalence of self-reported acute symptoms by severity from pre-infection to follow-up over a 1-month period among adults in influenza high-risk group (all ages)

| **Acute symptoms** | **Time of Survey** | | | | | | | | | | | |
| --- | --- | --- | --- | --- | --- | --- | --- | --- | --- | --- | --- | --- |
|  | **Prior to infection**  **(N=225)** | **Worst moment**  **(N=225)** | **Day 1**  **(N=225)** | **Day 2**  **(N=192)** | **Day 3 (N=191)** | **Day 4**  **(N=186)** | **Day 5**  **(N=184)** | **Day 6**  **(N=180)** | **Day 7**  **(N=181)** | **Day 10**  **(N=173)** | **Day 14**  **(N=171)** | **Week 4**  **(N=165)** |
| **Any symptoms, %** |  |  |  |  |  |  |  |  |  |  |  |  |
| stuffy or runny nose | 30.7 | 96.0 | 97.8 | 90.6 | 78.0 | 80.1 | 71.7 | 65.6 | 66.3 | 68.8 | 55.0 | 39.4 |
| cough | 19.1 | 98.7 | 98.7 | 96.9 | 95.3 | 90.9 | 87.0 | 84.4 | 82.3 | 72.8 | 64.3 | 38.2 |
| fatigue or tiredness | 24.9 | 96.9 | 95.1 | 87.0 | 74.9 | 65.6 | 63.6 | 53.3 | 53.0 | 44.5 | 33.9 | 23.6 |
| headache | 18.7 | 89.3 | 84.9 | 63.5 | 45.0 | 39.3 | 37.5 | 33.3 | 33.7 | 26.0 | 21.1 | 20.6 |
| body aches | 22.2 | 92.4 | 85.3 | 65.1 | 50.3 | 42.5 | 32.1 | 29.4 | 28.2 | 23.7 | 24.0 | 20.6 |
| feeling hot or feverish | 1.3 | 90.2 | 84.0 | 44.8 | 25.1 | 17.2 | 11.4 | 10.0 | 9.4 | 4.1 | 3.5 | 3.0 |
| chills or shivering | 2.7 | 89.3 | 81.8 | 32.8 | 14.7 | 9.1 | 8.7 | 6.1 | 5.0 | 2.9 | 1.2 | 1.2 |
| sore throat | 6.7 | 81.3 | 78.2 | 58.3 | 43.5 | 37.6 | 29.9 | 23.3 | 20.4 | 17.3 | 12.3 | 7.3 |
| difficulty breathing | 7.1 | 68.9 | 67.1 | 46.4 | 40.3 | 33.3 | 28.3 | 23.3 | 24.9 | 23.1 | 18.7 | 8.5 |
| nausea | 3.6 | 50.2 | 47.1 | 27.1 | 18.9 | 12.4 | 12.5 | 11.1 | 8.8 | 7.5 | 3.5 | 1.2 |
| vomit | 0.4 | 20.0 | 15.6 | 2.6 | 4.2 | 1.1 | 2.2 | 3.3 | 0.6 | 2.3 | 0.0 | 1.8 |
| diarrhea | 4.9 | 42.7 | 37.8 | 32.8 | 28.8 | 28.0 | 21.2 | 15.6 | 16.6 | 15.0 | 8.2 | 4.9 |
| **Moderate-to-severe, %** |  |  |  |  |  |  |  |  |  |  |  |  |
| stuffy or runny nose | 0.9 | 78.7 | 72.0 | 42.7 | 25.7 | 21.0 | 17.9 | 13.9 | 14.9 | 11.0 | 5.9 | 4.2 |
| cough | 1.8 | 88.4 | 80.4 | 56.3 | 45.6 | 33.9 | 28.8 | 23.3 | 25.4 | 23.1 | 8.8 | 1.2 |
| fatigue or tiredness | 1.8 | 86.2 | 75.6 | 45.3 | 29.8 | 24.2 | 16.9 | 11.7 | 9.4 | 5.2 | 4.1 | 2.4 |
| headache | 2.7 | 73.3 | 61.8 | 27.1 | 12.6 | 11.8 | 10.9 | 9.4 | 7.2 | 6.4 | 4.1 | 2.4 |
| body aches | 0.9 | 77.8 | 65.3 | 25.0 | 10.0 | 10.2 | 7.6 | 7.2 | 5.0 | 3.5 | 2.9 | 4.2 |
| feeling hot or feverish | 0.4 | 78.7 | 60.9 | 15.1 | 4.7 | 5.4 | 2.7 | 1.7 | 1.1 | 0.0 | 0.6 | 0.6 |
| chills or shivering | 0.0 | 74.2 | 58.7 | 10.9 | 1.6 | 1.6 | 0.5 | 1.1 | 0.0 | 0.0 | 0.6 | 0.6 |
| sore throat | 0.0 | 59.6 | 43.1 | 22.9 | 10.0 | 6.5 | 6.5 | 4.4 | 4.4 | 1.2 | 0.6 | 0.6 |
| difficulty breathing | 0.4 | 38.2 | 28.9 | 12.0 | 7.3 | 5.9 | 7.6 | 4.4 | 4.4 | 5.8 | 1.2 | 1.2 |
| nausea | 0.0 | 24.9 | 12.0 | 6.3 | 3.7 | 2.7 | 1.1 | 2.8 | 0.0 | 0.6 | 0.6 | 0.0 |
| vomit | 0.0 | 5.8 | 4.9 | 0.0 | 0.0 | 0.0 | 0.5 | 0.6 | 0.0 | 0.0 | 0.0 | 0.0 |
| diarrhea | 0.4 | 14.7 | 12.9 | 7.8 | 2.6 | 4.8 | 2.7 | 2.2 | 1.1 | 0.6 | 0.6 | 0.6 |
| **Severe, %** |  |  |  |  |  |  |  |  |  |  |  |  |
| stuffy or runny nose | 0.0 | 39.6 | 25.3 | 9.9 | 3.1 | 2.7 | 3.8 | 1.7 | 0.6 | 1.2 | 0.0 | 0.0 |
| cough | 0.0 | 57.3 | 42.2 | 14.1 | 8.9 | 8.1 | 4.4 | 3.9 | 2.8 | 2.9 | 1.8 | 0.0 |
| fatigue or tiredness | 0.4 | 54.2 | 39.6 | 10.4 | 4.7 | 3.8 | 1.6 | 0.0 | 1.1 | 0.6 | 0.0 | 0.0 |
| headache | 0.0 | 43.6 | 29.8 | 6.8 | 2.1 | 1.6 | 3.3 | 1.1 | 1.1 | 0.6 | 1.2 | 0.0 |
| body aches | 0.0 | 42.7 | 26.2 | 4.2 | 1.6 | 1.6 | 0.5 | 0.0 | 0.0 | 1.2 | 0.6 | 0.0 |
| feeling hot or feverish | 0.4 | 36.9 | 24.9 | 3.1 | 1.1 | 0.0 | 0.5 | 0.0 | 0.0 | 0.0 | 0.0 | 0.0 |
| chills or shivering | 0.0 | 44.4 | 27.6 | 2.6 | 0.5 | 0.0 | 0.5 | 0.0 | 0.0 | 0.0 | 0.0 | 0.0 |
| sore throat | 0.0 | 32.0 | 20.4 | 3.7 | 3.1 | 0.5 | 1.6 | 1.1 | 0.0 | 0.0 | 0.0 | 0.0 |
| difficulty breathing | 0.0 | 11.6 | 6.2 | 1.0 | 2.1 | 1.6 | 2.2 | 1.7 | 0.6 | 0.6 | 0.0 | 0.0 |
| nausea | 0.0 | 4.4 | 2.2 | 0.5 | 0.5 | 0.0 | 0.5 | 0.0 | 0.0 | 0.0 | 0.0 | 0.0 |
| vomit | 0.0 | 1.8 | 0.9 | 0.0 | 0.0 | 0.0 | 0.5 | 0.0 | 0.0 | 0.0 | 0.0 | 0.0 |
| diarrhea | 0.0 | 4.4 | 3.6 | 1.0 | 1.1 | 0.5 | 1.1 | 0.0 | 0.0 | 0.0 | 0.0 | 0.0 |

**Table S7**. Prevalence of self-reported acute symptoms by severity from pre-infection to follow-up over a 1-month period among adults in influenza high-risk group <65 years

| **Acute symptoms** | **Time of Survey** | | | | | | | | | | | |
| --- | --- | --- | --- | --- | --- | --- | --- | --- | --- | --- | --- | --- |
|  | **Prior to infection**  **(N=188)** | **Worst moment**  **(N=188)** | **Day 1**  **(N=188)** | **Day 2**  **(N=159)** | **Day 3 (N=158)** | **Day 4**  **(N=155)** | **Day 5**  **(N=150)** | **Day 6**  **(N=148)** | **Day 7**  **(N=148)** | **Day 10**  **(N=141)** | **Day 14**  **(N=139)** | **Week 4**  **(N=133)** |
| **Mild-to-severe, %** |  |  |  |  |  |  |  |  |  |  |  |  |
| stuffy or runny nose | 31.4 | 97.3 | 98.9 | 93.1 | 79.1 | 82.6 | 73.3 | 68.2 | 68.2 | 69.5 | 56.8 | 38.4 |
| cough | 17.0 | 98.9 | 98.9 | 96.9 | 94.9 | 90.3 | 86.7 | 85.8 | 83.1 | 70.9 | 62.6 | 36.1 |
| fatigue or tiredness | 26.1 | 96.8 | 95.2 | 88.1 | 77.9 | 67.1 | 65.3 | 56.1 | 56.1 | 48.2 | 35.3 | 23.3 |
| headache | 19.7 | 91.0 | 86.7 | 67.3 | 46.2 | 40.7 | 40.0 | 35.8 | 37.8 | 30.5 | 24.5 | 19.6 |
| body aches | 21.8 | 94.2 | 88.3 | 66.0 | 51.9 | 43.9 | 33.3 | 33.1 | 29.7 | 25.5 | 25.9 | 18.8 |
| feeling hot or feverish | 1.6 | 92.0 | 87.2 | 45.9 | 27.2 | 18.1 | 12.7 | 11.5 | 10.8 | 5.0 | 4.3 | 3.0 |
| chills or shivering | 2.7 | 92.0 | 84.0 | 35.9 | 17.1 | 9.7 | 8.7 | 6.1 | 4.7 | 2.8 | 1.4 | 1.5 |
| sore throat | 6.4 | 84.0 | 80.3 | 59.1 | 43.7 | 37.4 | 30.7 | 23.7 | 20.3 | 18.4 | 13.0 | 8.3 |
| difficulty breathing | 6.9 | 71.8 | 71.3 | 50.3 | 43.0 | 35.5 | 29.3 | 25.7 | 26.4 | 24.1 | 19.4 | 8.3 |
| nausea | 4.3 | 51.1 | 48.9 | 27.7 | 18.4 | 12.3 | 12.7 | 12.2 | 8.8 | 7.8 | 3.6 | 1.5 |
| vomit | 0.0 | 22.3 | 18.1 | 3.1 | 4.4 | 1.3 | 2.7 | 4.1 | 0.7 | 2.1 | 0.0 | 2.3 |
| diarrhea | 4.3 | 44.7 | 38.3 | 34.0 | 31.0 | 30.3 | 22.7 | 16.9 | 18.2 | 16.3 | 9.4 | 5.3 |
| **Moderate-to-severe, %** |  |  |  |  |  |  |  |  |  |  |  |  |
| stuffy or runny nose | 1.1 | 81.4 | 75.5 | 44.7 | 26.0 | 21.3 | 18.0 | 14.9 | 15.5 | 11.4 | 6.5 | 2.3 |
| cough | 1.1 | 90.4 | 81.4 | 56.6 | 47.5 | 34.8 | 29.3 | 24.3 | 26.4 | 23.4 | 10.1 | 1.5 |
| fatigue or tiredness | 2.1 | 88.8 | 79.8 | 49.1 | 30.4 | 26.5 | 17.3 | 12.8 | 8.8 | 5.0 | 4.3 | 2.3 |
| headache | 3.2 | 75.0 | 66.5 | 30.2 | 12.7 | 11.6 | 12.7 | 10.8 | 7.4 | 7.1 | 4.3 | 3.0 |
| body aches | 1.1 | 81.9 | 71.8 | 27.0 | 10.1 | 10.3 | 7.3 | 8.1 | 5.4 | 2.8 | 2.9 | 3.8 |
| feeling hot or feverish | 0.5 | 83.0 | 65.4 | 16.4 | 4.4 | 5.2 | 3.3 | 2.0 | 1.4 | 0.0 | 0.7 | 0.8 |
| chills or shivering | 0.0 | 77.7 | 63.3 | 12.0 | 1.9 | 1.9 | 0.7 | 1.4 | 0.0 | 0.0 | 0.7 | 0.8 |
| sore throat | 0.0 | 60.6 | 44.2 | 22.6 | 8.9 | 6.5 | 7.3 | 5.4 | 4.7 | 1.4 | 0.7 | 0.8 |
| difficulty breathing | 0.0 | 41.0 | 30.9 | 13.8 | 7.0 | 6.5 | 8.7 | 5.4 | 5.4 | 6.4 | 1.4 | 1.5 |
| nausea | 0.0 | 26.1 | 13.3 | 6.9 | 3.2 | 1.9 | 1.3 | 2.0 | 0.0 | 0.0 | 0.0 | 0.0 |
| vomit | 0.0 | 6.4 | 5.9 | 0.0 | 0.0 | 0.0 | 0.7 | 0.7 | 0.0 | 0.0 | 0.0 | 0.0 |
| diarrhea | 0.5 | 15.4 | 13.8 | 8.2 | 2.5 | 5.8 | 3.3 | 2.7 | 1.4 | 0.7 | 0.7 | 0.8 |
| **Severe, %** |  |  |  |  |  |  |  |  |  |  |  |  |
| stuffy or runny nose | 0.0 | 43.1 | 28.7 | 10.1 | 3.8 | 2.6 | 4.7 | 2.0 | 0.7 | 1.4 | 0.0 | 0.0 |
| cough | 0.0 | 59.6 | 45.2 | 13.8 | 9.5 | 9.0 | 5.3 | 4.7 | 3.4 | 3.6 | 2.2 | 0.0 |
| fatigue or tiredness | 0.5 | 58.0 | 44.2 | 12.0 | 5.1 | 3.9 | 2.0 | 0.0 | 1.4 | 0.7 | 0.0 | 0.0 |
| headache | 0.0 | 49.5 | 35.1 | 8.2 | 2.5 | 1.9 | 4.0 | 1.4 | 1.4 | 0.7 | 1.4 | 0.0 |
| body aches | 0.0 | 47.9 | 30.9 | 5.0 | 1.9 | 1.9 | 0.7 | 0.0 | 0.0 | 1.4 | 0.7 | 0.0 |
| feeling hot or feverish | 0.5 | 41.0 | 27.7 | 3.8 | 1.3 | 0.0 | 0.7 | 0.0 | 0.0 | 0.0 | 0.0 | 0.0 |
| chills or shivering | 0.0 | 48.9 | 30.9 | 3.1 | 0.6 | 0.0 | 0.7 | 0.0 | 0.0 | 0.0 | 0.0 | 0.0 |
| sore throat | 0.0 | 34.6 | 22.9 | 3.1 | 3.2 | 0.7 | 2.0 | 1.4 | 0.0 | 0.0 | 0.0 | 0.0 |
| difficulty breathing | 0.0 | 13.3 | 7.5 | 1.3 | 2.5 | 1.9 | 2.7 | 2.0 | 0.7 | 0.7 | 0.0 | 0.0 |
| nausea | 0.0 | 5.3 | 2.7 | 0.6 | 0.6 | 0.0 | 0.7 | 0.0 | 0.0 | 0.0 | 0.0 | 0.0 |
| vomit | 0.0 | 1.6 | 1.1 | 0.0 | 0.0 | 0.0 | 0.7 | 0.0 | 0.0 | 0.0 | 0.0 | 0.0 |
| diarrhea | 0.0 | 43.1 | 28.7 | 10.1 | 3.8 | 2.6 | 4.7 | 2.0 | 0.7 | 1.4 | 0.0 | 0.0 |

**Table S8**. Prevalence of self-reported symptoms by severity from pre-infection to follow-up over a 1-month period among adults in COVID-19 high-risk group (All ages)

| Symptom by severity | Time of Survey | | | | | | | | | | | |
| --- | --- | --- | --- | --- | --- | --- | --- | --- | --- | --- | --- | --- |
|  | Prior to infection  (N=352) | Worst moment  (N=352) | Day 1  (N=352) | Day 2  (N=300) | Day 3 (N=295) | Day 4  (N=287) | Day 5  (N=287) | Day 6  (N=279) | Day 7  (N=280) | Day 10  (N=262) | Day 14  (N=258) | Week 4  (N=250) |
| **Any symptoms, %** |  |  |  |  |  |  |  |  |  |  |  |  |
| stuffy or runny nose | 28.3 | 96.9 | 97.8 | 91.5 | 83.3 | 79.3 | 70.3 | 67.6 | 66.8 | 62.6 | 53.1 | 37.3 |
| cough | 18.1 | 98.1 | 97.2 | 93.5 | 92.7 | 89.0 | 85.9 | 82.6 | 80.6 | 70.2 | 58.5 | 33.3 |
| fatigue or tiredness | 27.2 | 97.8 | 96.7 | 88.2 | 77.0 | 66.9 | 62.1 | 52.3 | 48.4 | 43.0 | 33.9 | 23.8 |
| headache | 21.1 | 90.3 | 88.1 | 66.0 | 47.7 | 39.7 | 35.9 | 30.6 | 32.9 | 24.2 | 17.7 | 18.7 |
| body aches | 19.7 | 93.6 | 87.8 | 65.4 | 50.0 | 38.6 | 28.6 | 27.1 | 23.7 | 19.3 | 18.5 | 17.9 |
| feeling hot or feverish | 1.1 | 91.1 | 86.7 | 44.4 | 23.7 | 14.8 | 10.3 | 9.6 | 6.7 | 4.5 | 2.3 | 2.4 |
| chills or shivering | 2.5 | 90.6 | 83.3 | 34.3 | 15.3 | 7.9 | 6.9 | 4.6 | 3.9 | 2.3 | 1.2 | 1.2 |
| sore throat | 6.7 | 84.4 | 81.1 | 60.1 | 43.3 | 37.6 | 28.3 | 22.8 | 19.4 | 17.0 | 10.8 | 7.9 |
| difficulty breathing | 6.1 | 67.2 | 65.8 | 45.1 | 37.3 | 32.8 | 25.9 | 20.3 | 20.1 | 18.5 | 15.4 | 7.1 |
| nausea | 5.3 | 51.7 | 47.8 | 26.1 | 19.7 | 13.8 | 11.7 | 8.5 | 8.5 | 6.4 | 3.5 | 2.4 |
| vomit | 0.6 | 19.4 | 15.0 | 4.6 | 3.7 | 0.7 | 1.7 | 2.1 | 0.7 | 1.5 | 0.0 | 2.0 |
| diarrhea | 4.2 | 41.1 | 36.4 | 29.7 | 26.3 | 23.5 | 19.3 | 13.5 | 14.5 | 13.2 | 8.1 | 6.8 |
| **Moderate-to-severe, %** |  |  |  |  |  |  |  |  |  |  |  |  |
| stuffy or runny nose | 1.7 | 79.4 | 73.9 | 44.8 | 27.7 | 19.3 | 16.2 | 13.5 | 13.1 | 9.8 | 5.8 | 4.4 |
| cough | 1.9 | 86.9 | 80.8 | 56.5 | 43.3 | 32.8 | 27.9 | 22.1 | 22.3 | 18.9 | 8.1 | 1.2 |
| fatigue or tiredness | 1.7 | 89.2 | 79.7 | 46.4 | 29.7 | 23.5 | 15.9 | 11.7 | 10.3 | 5.7 | 3.1 | 2.0 |
| headache | 2.2 | 74.4 | 62.8 | 27.1 | 14.3 | 11.7 | 9.0 | 8.5 | 6.0 | 6.0 | 3.5 | 2.4 |
| body aches | 0.6 | 79.2 | 67.5 | 24.8 | 9.3 | 8.3 | 5.2 | 5.3 | 3.5 | 3.0 | 2.3 | 3.6 |
| feeling hot or feverish | 0.3 | 80.8 | 64.4 | 14.4 | 5.3 | 3.8 | 1.7 | 1.1 | 0.7 | 0.8 | 0.4 | 0.8 |
| chills or shivering | 0.0 | 76.4 | 61.1 | 11.1 | 2.0 | 1.0 | 0.3 | 0.7 | 0.0 | 0.4 | 0.4 | 0.4 |
| sore throat | 0.0 | 61.1 | 46.4 | 21.2 | 10.3 | 6.6 | 5.9 | 4.3 | 3.9 | 1.9 | 0.4 | 0.4 |
| difficulty breathing | 0.3 | 34.7 | 26.4 | 11.8 | 7.0 | 4.5 | 6.9 | 4.3 | 3.9 | 4.5 | 1.2 | 0.8 |
| nausea | 0.3 | 24.7 | 13.3 | 6.9 | 3.3 | 2.1 | 1.4 | 2.1 | 0.4 | 0.8 | 0.8 | 0.4 |
| vomit | 0.3 | 5.8 | 4.7 | 0.3 | 0.0 | 0.0 | 0.3 | 0.4 | 0.0 | 0.0 | 0.0 | 0.0 |
| diarrhea | 0.6 | 14.4 | 13.3 | 6.9 | 3.0 | 4.1 | 3.1 | 2.1 | 1.1 | 0.8 | 0.8 | 0.4 |
| **Severe, %** |  |  |  |  |  |  |  |  |  |  |  |  |
| stuffy or runny nose | 0.3 | 41.1 | 26.1 | 9.8 | 3.3 | 2.4 | 2.4 | 1.1 | 0.7 | 1.1 | 0.0 | 0.0 |
| cough | 0.6 | 55.8 | 40.6 | 14.1 | 8.0 | 6.9 | 3.5 | 2.9 | 2.1 | 1.9 | 1.2 | 0.0 |
| fatigue or tiredness | 0.3 | 56.4 | 40.6 | 11.1 | 3.7 | 3.5 | 2.1 | 0.7 | 1.4 | 1.1 | 0.4 | 0.0 |
| headache | 0.0 | 43.6 | 30.3 | 6.2 | 2.3 | 1.0 | 2.1 | 1.1 | 1.4 | 0.4 | 0.8 | 0.0 |
| body aches | 0.0 | 45.6 | 28.3 | 3.6 | 1.7 | 1.0 | 0.3 | 0.0 | 0.4 | 1.1 | 0.4 | 0.0 |
| feeling hot or feverish | 0.3 | 41.7 | 26.4 | 3.6 | 0.7 | 0.0 | 0.3 | 0.0 | 0.0 | 0.4 | 0.0 | 0.0 |
| chills or shivering | 0.0 | 44.7 | 28.3 | 3.3 | 0.3 | 0.0 | 0.3 | 0.0 | 0.0 | 0.4 | 0.0 | 0.0 |
| sore throat | 0.0 | 33.1 | 19.7 | 5.2 | 2.7 | 1.0 | 1.4 | 1.1 | 0.4 | 0.0 | 0.0 | 0.0 |
| difficulty breathing | 0.0 | 10.3 | 5.3 | 1.3 | 1.7 | 1.0 | 1.4 | 1.1 | 0.4 | 0.4 | 0.0 | 0.0 |
| nausea | 0.0 | 6.7 | 2.5 | 0.7 | 0.3 | 0.3 | 0.3 | 0.0 | 0.0 | 0.0 | 0.0 | 0.0 |
| vomit | 0.3 | 1.7 | 1.1 | 0.3 | 0.0 | 0.0 | 0.3 | 0.0 | 0.0 | 0.0 | 0.0 | 0.0 |
| diarrhea | 0.3 | 5.3 | 4.2 | 1.3 | 0.7 | 1.0 | 1.0 | 0.0 | 0.0 | 0.0 | 0.0 | 0.0 |

COVID-19=coronavirus disease 2019.

**Table S9**. Summary of least-square estimates ^a^ of mean change in symptom measures from prior to infection among adults <65 at high risk for influenza complications and adults all ages at high risk for COVID-19 complications.

| **Time** | **Retention rate n (%)** | **Composite acute symptom score** | | **Number of any symptoms** | | **Number of moderate-to-severe acute symptoms** | | **Number of severe acute symptoms** | |
| --- | --- | --- | --- | --- | --- | --- | --- | --- | --- |
|  |  | **LSE (SE)** | **ES ^b^** | **LSE (SE)** | **ES ^b^** | **LSE (SE)** | **ES ^b^** | **LSE (SE)** | **ES ^b^** |
| **Influenza high-risk group <65 years** | | | | | | | | | |
| Prior to infection ^c^ | 188 (100.0) | 1.5 (1.8) |  | 1.4 (1.6) |  | 0.1 (0.3) |  | 0.0 (0.1) |  |
| Worst Moment | 188 (100.0) | **19.5 (0.4)^d^** | 3.37 | **8.1 (0.2)^d^** | 3.77 | **7.2 (0.2)^d^** | 3.11 | **4.1 (0.1)^d^** | 1.53 |
| Day 1 | 188 (100.0) | **16.7 (0.5)^d^** | 2.73 | **7.7 (0.2)^d^** | 3.30 | **6.1 (0.2)^d^** | 2.27 | **2.8 (0.1)^d^** | 1.18 |
| Day 2 | 159 (84.6) | **8.9 (0.4)^d^** | 1.60 | **5.4 (0.2)^d^** | 1.97 | **2.8 (0.2)^d^** | 1.16 | **0.6 (0.1)^d^** | 0.43 |
| Day 3 | 158 (84.0) | **6.2 (0.4)^d^** | 1.33 | **4.2 (0.2)^d^** | 1.56 | **1.6 (0.1)^d^** | 0.85 | **0.4 (0.1)^d^** | 0.40 |
| Day 4 | 155 (82.4) | **5.0 (0.3)^d^** | 1.15 | **3.5 (0.2)^d^** | 1.32 | **1.3 (0.1)^d^** | 0.72 | **0.2 (0.1)^d^** | 0.38 |
| Day 5 | 150 (79.8) | **4.3 (0.3)^d^** | 0.90 | **2.9 (0.2)^d^** | 1.11 | **1.1 (0.1)^d^** | 0.59 | **0.3 (0.1)^d^** | 0.25 |
| Day 6 | 148 (78.7) | **3.6 (0.3)^d^** | 0.86 | **2.6 (0.2)^d^** | 0.98 | **0.9 (0.1)^d^** | 0.52 | 0.2 (0.1) | 0.31 |
| Day 7 | 148 (78.7) | **3.2 (0.3)^d^** | 0.91 | **2.4 (0.2)^d^** | 0.96 | **0.7 (0.1)^d^** | 0.58 | 0.1 (0.1) | 0.25 |
| Day 10 | 141 (75.0) | **2.6 (0.3)^d^** | 0.81 | **2.0 (0.2)^d^** | 0.81 | **0.5 (0.1)^d^** | 0.52 | 0.1 (0.1) | 0.26 |
| Day 14 | 139 (73.9) | **1.7 (0.3)^d^** | 0.61 | **1.3 (0.2)^d^** | 0.61 | **0.3 (0.1)^d^** | 0.32 | 0.1 (0.1) | 0.29 |
| Week 4 | 133 (70.7) | **0.7 (0.2)^d^** | 0.25 | **0.5 (0.2)^d^** | 0.23 | **0.1 (0.1)^d^** | 0.20 | 0.0 (0.1) | 0.32 |
| **COVID-19 high-risk group (All ages)** | | | | | | | | | |
| Prior to infection ^c^ | 352 (100.0) | 1.5 (1.8) |  | 1.4 (1.5) |  | 0.1 (0.4) |  | 0.0 (0.2) |  |
| Worst Moment | 352 (100.0) | **19.1 (0.3)^d^** | 3.21 | **8.1 (0.1)^d^** | 3.82 | **7.1 (0.1)^d^** | 2.96 | **3.9 (0.1)^d^** | 1.46 |
| Day 1 | 352 (100.0) | **16.2 (0.4)^d^** | 2.63 | **7.7 (0.1)^d^** | 3.32 | **6.0 (0.1)^d^** | 2.21 | **2.6 (0.1)^d^** | 1.10 |
| Day 2 | 300 (85.2) | **8.6 (0.3)^d^** | 1.56 | **5.3 (0.2)^d^** | 1.94 | **2.7 (0.1)^d^** | 1.10 | **0.6 (0.1)^d^** | 0.45 |
| Day 3 | 295 (83.8) | **5.9 (0.3)^d^** | 1.31 | **4.1 (0.2)^d^** | 1.55 | **1.6 (0.1)^d^** | 0.82 | **0.3 (0.1)^d^** | 0.35 |
| Day 4 | 287 (81.5) | **4.7 (0.3)^d^** | 1.15 | **3.3 (0.2)^d^** | 1.33 | **1.2 (0.1)^d^** | 0.71 | **0.2 (0.1)^d^** | 0.35 |
| Day 5 | 287 (81.5) | **3.8 (0.3)^d^** | 0.93 | **2.7 (0.2)^d^** | 1.09 | **0.9 (0.1)^d^** | 0.59 | 0.2(0.1) | 0.22 |
| Day 6 | 279 (79.3) | **3.1 (0.3)^d^** | 0.84 | **2.3 (0.2)^d^** | 0.93 | **0.8 (0.1)^d^** | 0.53 | 0.1 (0.1) | 0.28 |
| Day 7 | 280 (79.5) | **2.8 (0.2)^d^** | 0.84 | **2.1 (0.2)^d^** | 0.88 | **0.6 (0.1)^d^** | 0.55 | 0.1 (0.1) | 0.23 |
| Day 10 | 262 (74.4) | **2.3 (0.2)^d^** | 0.70 | **1.7 (0.2)^d^** | 0.73 | **0.5 (0.1)^d^** | 0.45 | 0.1 (0.1) | 0.25 |
| Day 14 | 258 (73.3) | **1.4 (0.2)^d^** | 0.53 | **1.1 (0.1)^d^** | 0.53 | **0.2 (0.1)^d^** | 0.29 | 0.1 (0.1) | 0.24 |
| Week 4 | 250 (71.0) | **0.7 (0.2)^d^** | 0.29 | **0.5 (0.1)^d^** | 0.26 | **0.2 (0.1)^d^** | 0.23 | 0.0 (0.1) | 0.26 |

COVID-19=coronavirus disease 2019; LSE=least square estimate; SE=standard error; ES=effect size.

^a^ Least square estimates based on mixed models for repeated measures, which include covariates of time, High Risk, gender, race\ethnicity, region, Social Vulnerability Index, anti-viral use, ≥1 comorbidity, receiving influenza vaccine, baseline scores for each outcome, and interaction terms of time*High Risk, time*≥1 comorbidity, and time*receiving influenza vaccine, with unstructured correlation matrix for repeated measures.

^b^ ES refers to within-cohort effect size and was calculated as the least square estimate of mean change scores divided by the observed standard deviation of change scores from prior to infection to follow-up. These standardized effect sizes were interpreted using Cohen’s conventions, with threshold values of 0.2 to <0.5, 0.5 to <0.8, and ≥0.8 indicating “small,” “medium,” and “large” effects, respectively.

^c^ Mean (standard deviation) of observed scores prior to infection were reported.

^d^ *P*<0.05 considered statistically significant.

**Table S10**. Reasons for attrition

| **Reason** | **N (%)** |
| --- | --- |
| I was sick and didn’t feel well enough to answer | 14 (11.6) |
| My illness-related symptoms resolved and I preferred to stop answering illness-related questions | 1 (0.8) |
| The survey was too long | 3 (2.5) |
| I was too busy to complete it or forgot | 74 (61.2) |
| I had technical difficulties accessing or opening the link to the survey | 16 (13.2) |
| The compensation amount was not enough | 2 (1.7) |
| Other | 11 (9.1) |
| **Total, N** | 121 |

**Table S11.** Comparison of characteristics between participant who completed the study and those who dropped out.

| **Characteristics** | **Completed 4 weeks**  **(N=500)** | **Dropped out**  **(N=224)** | **P-value** |
| --- | --- | --- | --- |
| Age, years, mean (SD) | 42.5 (13.0) | 40.8 (13.0) | 0.098 |
| Sex, n (%) |  |  | 0.979 |
| Female | 371 (74.2) | 166 (74.1) |  |
| Male | 129 (25.8) | 58 (25.9) |  |
| Race/Ethnicity, n (%) |  |  | **0.035** |
| White | 419 (83.8) | 167 (74.6) |  |
| Black or African American | 22 (4.4) | 13 (5.8) |  |
| Hispanic or Latino | 27 (5.4) | 25 (11.2) |  |
| Asian | 15 (3.0) | 9 (4.0) |  |
| Other | 17 (3.4) | 10 (4.5) |  |
| US Region of residence, n (%) |  |  | 0.607 |
| Northeast | 85 (17.0) | 43 (19.2) |  |
| South | 236 (47.2) | 102 (45.5) |  |
| Midwest | 151 (30.2) | 70 (31.3) |  |
| West | 26 (5.2) | 7 (3.1) |  |
| Other\Unknown | 2 (0.4) | 2 (0.9) |  |
| Employment status, n (%) |  |  | 0.461 |
| Yes | 406 (81.2) | 187 (83.5) |  |
| Social Vulnerability Index ^a^, mean (SD) | 0.31 (0.18) | 0.34 (0.20) | **0.028** |
| Social Vulnerability Index category, n (%) |  |  | 0.092 |
| <0.25 | 228 (45.6) | 87 (38.8) |  |
| ≥0.25 and <0.5 | 201 (40.2) | 92 (41.1) |  |
| ≥0.5 and <0.75 | 57 (11.4) | 33 (14.7) |  |
| ≥0.75 | 13 (2.6) | 12 (5.4) |  |
| Antiviral use, n (%) | 428 (85.6) | 188 (83.9) | 0.560 |
| Received the 2024-2025 influenza vaccine, n (%) | 261 (52.2) | 96 (42.9) | **0.020** |
| Pregnant, n (%) |  |  | 0.247 |
| No | 361 (72.2) | 159 (71.0) |  |
| Yes | 4 (0.8) | 5 (2.2) |  |
| Not applicable | 135 (27.0) | 60 (26.8) |  |
| Underlying health conditions, n (%) |  |  |  |
| Any current/active cancers or malignancies (other than skin cancer) | 2 (0.4) | 3 (1.3) | 0.158 |
| Cerebrovascular disease | 8 (1.6) | 0 (0.0) | 0.057 |
| Chronic kidney disease | 2 (0.4) | 1 (0.4) | 0.928 |
| Chronic lung conditions | 38 (7.6) | 13 (5.8) | 0.383 |
| Chronic liver disease | 7 (1.4) | 4 (1.8) | 0.695 |
| Endocrine disorders | 28 (5.6) | 5 (2.2) | **0.045** |
| Heart conditions | 17 (3.4) | 4 (1.8) | 0.232 |
| Obesity: BMI>40 | 75 (15.0) | 29 (12.9) | 0.467 |
| Weakened immune system/ immunocompromised | 12 (2.4) | 5 (2.2) | 0.890 |
| Blood disorders | 3 (0.6) | 2 (0.9) | 0.660 |
| Active Tuberculosis |  |  |  |
| Number of comorbidities ^b^, mean (SD) | 0.38 (0.69) | 0.29 (0.58) | 0.091 |
| ≥1 comorbidity ^b^, n (%) | 146 (29.2) | 54 (24.1) | 0.157 |
| ≥1 comorbidity or pregnant, n (%) | 150 (30.0) | 58 (25.9) | 0.259 |

SD=standard deviation; US=United States.

^a^ American Indian or Alaska Native, Native Hawaiian or Other Pacific Islanders, Patient Refused, Other, Unknown.

Bolded p-values indicate statistical significance at p <0.05.

**Table S12.** Patient characteristics of analysis cohort over time

|  | **Day 1**  **(N=724)** | **Day 7**  **(N=562)** | **Day 10**  **(N=522)** | **Day 14**  **(N=515)** | **Week 4**  **(N=500)** | **p-value** |
| --- | --- | --- | --- | --- | --- | --- |
| Age, years, mean (SD) | 42.0 (13.0) | 42.4 (13.0) | 42.4 (13.0) | 42.5 (13.0) | 42.5 (13.0) | 0.941 |
| Age group, n (%) |  |  |  |  |  | 1.000 |
| 18-29 | 147 (20.3) | 103 (18.3) | 96 (18.4) | 94 (18.3) | 91 (18.2) |  |
| 30-49 | 366 (50.6) | 293 (52.1) | 273 (52.3) | 270 (52.4) | 263 (52.6) |  |
| 50-64 | 174 (24.0) | 133 (23.7) | 121 (23.2) | 119 (23.1) | 114 (22.8) |  |
| 65-74 | 34 (4.7) | 30 (5.3) | 29 (5.6) | 29 (5.6) | 29 (5.8) |  |
| 75+ | 3 (0.4) | 3 (0.5) | 3 (0.6) | 3 (0.6) | 3 (0.6) |  |
| Gender, n (%) |  |  |  |  |  | 0.999 |
| Female | 537 (74.2) | 414 (73.7) | 388 (74.3) | 382 (74.2) | 371 (74.2) |  |
| Male | 187 (25.8) | 148 (26.3) | 134 (25.7) | 133 (25.8) | 129 (25.8) |  |
| Race/Ethnicity, n (%) |  |  |  |  |  | 0.999 |
| White or Caucasian | 586 (80.9) | 462 (82.2) | 436 (83.5) | 431 (83.7) | 419 (83.8) |  |
| Black or African American | 35 (4.8) | 28 (5.0) | 24 (4.6) | 23 (4.5) | 22 (4.4) |  |
| Hispanic | 52 (7.2) | 36 (6.4) | 29 (5.6) | 28 (5.4) | 27 (5.4) |  |
| Asian | 24 (3.3) | 17 (3.0) | 15 (2.9) | 15 (2.9) | 15 (3.0) |  |
| Other ^a^ | 27 (3.7) | 19 (3.4) | 18 (3.4) | 18 (3.5) | 17 (3.4) |  |
| US Region, n (%) |  |  |  |  |  | 1.000 |
| Northeast | 128 (17.7) | 96 (17.1) | 86 (16.5) | 86 (16.7) | 85 (17.0) |  |
| South | 338 (46.7) | 266 (47.3) | 249 (47.7) | 244 (47.4) | 236 (47.2) |  |
| Midwest | 221 (30.5) | 169 (30.1) | 158 (30.3) | 157 (30.5) | 151 (30.2) |  |
| West | 33 (4.6) | 29 (5.2) | 27 (5.2) | 26 (5.0) | 26 (5.2) |  |
| Other\Unknown | 4 (0.6) | 2 (0.4) | 2 (0.4) | 2 (0.4) | 2 (0.4) |  |
| Social Vulnerability Index, mean (SD) | 0.32 (0.19) | 0.32 (0.19) | 0.31 (0.18) | 0.31 (0.18) | 0.31 (0.18) | 0.896 |
| Number of comorbidities, mean (SD) | 0.71 (0.94) | 0.73 (0.95) | 0.73 (0.95) | 0.73 (0.95) | 0.73 (0.95) | 0.995 |

SD=standard deviation; US=United States.

^a^ American Indian or Alaska Native, Native Hawaiian or Other Pacific Islanders, Patient Refused, Other, Unknown.

**Table S13.** Comparisons of adults included and not included in the final analysis

| **Characteristic** | **Included**  **(N=724)** | **Not Included**  **(N=50003)** | **P value** |
| --- | --- | --- | --- |
| Age, years, mean (SD) | 42.0 (13.0) | 41.3 (15.4) | 0.236 |
| Age group, n (%) |  |  | **<0.001** |
| 18-29 | 147 (20.3) | 14,267 (28.5) |  |
| 30-49 | 366 (50.6) | 19,726 (39.4) |  |
| 50-64 | 174 (24.0) | 12,197 (24.4) |  |
| 65-74 | 34 (4.7) | 2,889 (5.8) |  |
| 75+ | 3 (0.4) | 924 (1.8) |  |
| Sex, n (%) |  |  | **<0.001** |
| Female | 537 (74.2) | 29,948 (59.9) |  |
| Male | 187 (25.8) | 20,004 (40.0) |  |
| Unknown | 0 (0.0) | 51 (0.1) |  |
| Race/Ethnicity, n (%) |  |  | **<0.001** |
| White or Caucasian | 552 (76.2) | 31,374 (62.7) |  |
| Black or African American | 40 (5.5) | 3,425 (6.8) |  |
| Hispanic | 48 (6.6) | 6,144 (12.3) |  |
| Asian | 20 (2.8) | 2,781 (5.6) |  |
| Other ^a^ | 64 (8.8) | 6,279 (12.6) |  |
| US Region, n (%) |  |  | **<0.001** |
| Northeast | 128 (17.7) | 6,762 (13.5) |  |
| South | 338 (46.7) | 29,006 (58.0) |  |
| Midwest | 221 (30.5) | 12,015 (24.0) |  |
| West | 33 (4.6) | 2,075 (4.1) |  |
| Other\Unknown | 4 (0.6) | 145 (0.3) |  |
| Insurance type, n (%) |  |  | 0.334 |
| Commercial | 525 (72.5) | 35,135 (70.3) |  |
| Government^b^ | 50 (6.9) | 4,389 (8.8) |  |
| Other | 0 (0.0) | 3 (0.0) |  |
| Self-pay | 138 (19.1) | 9,576 (19.2) |  |
| Missing | 11 | 900 |  |
| Social Vulnerability Index, mean (SD) | 0.32 (0.19) | 0.35 (0.20) | **0.002** |

SD=standard deviation; US=United States.

^a^ American Indian or Alaska Native, Native Hawaiian or Other Pacific Islander, Patient Refused, Other, Unknown.

^b^ Medicare, Medicaid, Government.

Note: Patient characteristics compared between those included vs. not included were retrieved from the ambulatory care clinic testing database, rather than from survey. Race and ethnicity may be reported differently. Insurance type was not asked in the survey.

Bolded p-values indicate statistical significance at p <0.05
